# Replicating superspreader dynamics with compartmental models

**DOI:** 10.1038/s41598-023-42567-3

**Published:** 2023-09-15

**Authors:** Michael T. Meehan, Angus Hughes, Romain R. Ragonnet, Adeshina I. Adekunle, James M. Trauer, Pavithra Jayasundara, Emma S. McBryde, Alec S. Henderson

**Affiliations:** 1 Australian Institute of Tropical Health and Medicine, James Cook University, Townsville, 4811 Australia; 2 College of Public Health, Medical and Veterinary Sciences, James Cook University, Townsville, 4811 Australia; 3 School of Public Health and Preventive Medicine, Monash University, Melbourne, 3800 Australia; 4 Defence Science and Technology Group, Department of Defence, Melbourne, 3207 Australia

**Keywords:** Computational models, Infectious diseases, Epidemiology

## Abstract

Infectious disease outbreaks often exhibit superspreader dynamics, where most infected people generate no, or few secondary cases, and only a small fraction of individuals are responsible for a large proportion of transmission. Although capturing this heterogeneity is critical for estimating outbreak risk and the effectiveness of group-specific interventions, it is typically neglected in compartmental models of infectious disease transmission—which constitute the most common transmission dynamic modeling framework. In this study we propose different classes of compartmental epidemic models that incorporate transmission heterogeneity, fit them to a number of real outbreak datasets, and benchmark their performance against the canonical superspreader model (i.e., the negative binomial branching process model). We find that properly constructed compartmental models can capably reproduce observed superspreader dynamics and we provide the pathogen-specific parameter settings required to do so. As a consequence, we also show that compartmental models parameterized according to a binary clinical classification have limited support.

## Introduction

Many infectious disease outbreaks are characterized by superspreading behavior, where individuals with high numbers of contacts, high levels of infectiousness, or both generate a disproportionately large number of secondary infections^1,2^. Recent outbreaks of severe acute respiratory syndrome coronavirus-2 (SARS-CoV-2)^3–5^, SARS-CoV-1^6,7^, Middle-East respiratory syndrome coronavirus (MERS-CoV)^8,9^ and Ebola virus (EBV)^10^ serve as prime examples, in which more than 80% of transmission was attributed to less than 20% of cases. Such highly skewed transmission distributions have important consequences for epidemic dynamics and control: making outbreaks less likely but more explosive, and targeted interventions towards high-risk groups exceedingly effective^11^.

To explain the observed heterogeneity in infectious disease transmission, Lloyd-Smith et al.^11^ popularized a branching process model under which the number of secondary cases, *Z*, generated by each infectious individual is a Poisson random variable with rate parameter $\nu$, where $\nu$ is an individual’s reproductive potential. The authors modelled $\nu$ using a Gamma distribution with population mean *R* (i.e., the reproductive number) and dispersion parameter *k*, resulting in a negative binomial distribution for the number of offspring *Z*. In this model, lower values of *k* correspond to higher levels of transmission heterogeneity: when $k=1$, the reproductive potential $\nu \sim \textrm{Exp}(1/R)$ (which is equivalent to the behavior of the standard Susceptible-Infected-Removed (SIR) compartmental model); whilst in the limit $k\rightarrow \infty$ all individuals have $\nu = R$. Fitting to past infectious disease outbreaks, the authors found substantial evidence for a high degree of individual variability ($k < 1$), indicating the general negative binomial model was overwhelmingly preferred over alternatives with $k\ge 1$. Following this, the negative binomial model has been widely adopted as the canonical model for analyzing heterogeneous transmission data, although several alternatives have been proposed (e.g., see Refs.^12,13^).

Whilst considerable overdispersion is a generic feature of aggregated transmission data (e.g., secondary case counts and cluster sizes), the actual time period for which individuals are infected typically follows a more homogeneous distribution with a positive (i.e., non-zero) mode^14,15^. This property strongly influences the temporal dynamics of transmission, and shapes the relationship between the epidemic growth rate and the reproductive number^16^. Consequently, temporal models must capture both the mean and shape of the infected period distribution to avoid negatively biasing estimates of the reproductive number and, in turn, the effort required to achieve control^17^.

Fortunately, realistic temporal dynamics can be readily recovered in compartmental epidemic models — the commonest approach to infectious disease modelling — using the method of stages^18^. Here individuals transition through several serial infective classes (i.e., compartments) throughout their infected lifetime, such that the total infected period follows a HypoExponential distribution with a positive mode^18,19^. Importantly, only those compartments that are actively infectious contribute to the reproductive potential distribution, with non-infectious compartments (e.g., (E)xposed compartments) only affecting temporal evolution. In the simplest case — where the transmission and removal rates are constant across each infective state — the reproductive potential $\nu$ is Erlang-distributed, thereby recovering a special case of Lloyd-Smith’s negative binomial model: one where *k* is an integer. The challenge then becomes reconciling highly overdispersed patterns of transmission ($k < 1$), with the more homogeneous temporal dynamics of infection ($k \ge 1$) within a single modelling framework.

One possible resolution is to use multi-type compartmental models, in which individuals are assigned to parallel infectious streams with varying characteristics. Transmission heterogeneity is achieved through differential infectiousness across each infectious stream (i.e., type), whilst temporal homogeneity is replicated through the method of stages (i.e., serial infectious compartments within each stream). Multi-type compartmental models have been used previously to capture population heterogeneity, with transmission potential typically linked to symptomatic status (see e.g., Ref.^20^). However, whilst symptomatic or clinical status may appear to be a reasonable surrogate for individual transmissibility, the extent to which this generates sufficient heterogeneity remains untested.

In this study, we design and evaluate compartmental models that attempt to simultaneously replicate transmission *heterogeneity* and temporal *homogeneity*. The general model is composed of two parallel streams of infective compartments (i.e., subspreaders and superspreaders), with each stream consisting of two infective compartments structured in series — thus allowing a postive mode for the infected period distribution. The general model is parameterized by: the population mean reproductive number, *R*; the relative transmission potential of the first and second serial compartments within each type, $\sigma$; the proportion of the population in the superspreader class, *c*; and the transmission potential of the subspreader class relative to that of superspreaders, $\rho$.

Within this general framework we also analyze a number of constrained sub-variants including: a “clinical” model in which the superspreader fraction is pre-determined by the pathogen-specific proportion of individuals that are symptomatic; a Susceptible-Exposed-Infectious-Recovered (SEIR) variant where the first serial compartment of each type is assumed non-infectious (i.e., $\sigma = 0$); and single-type variants of the above ($c=0$). A flow diagram of the general, unconstrained model is provided in Fig. S5 and a summary of the parameters specific to each sub-variant is given in Table 1. In addition to the baseline two-type model, we also analyze the behaviour of extended model architectures with greater than two types and varying numbers of serial compartments within each type (see Extended Analysis and Supplement).

**Table 1:** Parameter summary.

Type	Model	Fixed parameters	Free parameters ($\#$)
Benchmark	Negative binomial	—	$R, \, k\qquad (2)$
Two-type	General (2) (unconstrained)	—	*R*, $\sigma$, *c*, $\rho \qquad (4)$
	Clinical*	$c = c_{\textrm{symp}}$	*R*, $\sigma$, $\rho \qquad (3)$
	SEIR (2)	$\sigma = 0$	*R*, *c*, $\rho \qquad (3)$
Single-type	General (1) (unconstrained)	$c = 0$	*R*, $\sigma \qquad (2)$
	SEIR (1)	$\sigma =c=0$	$R\qquad (1)$

*For the clinical model, the superspreader fraction *c* is fixed by the pathogen-specific symptomatic fraction $c_{\textrm{symp}}$.

To investigate the performance of each candidate model and the range of best-fitting parameters we analyze secondary case count data from outbreaks of: EBV in Guinea^10,21^; MERS-CoV in the Republic of Korea^8,9^; Mpox virus in Zaire^22^; SARS-CoV-1 in Beijing^6^ and Singapore^7^; SARS-CoV-2 in China^5,23^, Hong Kong^3^, India^24^, Indonesia^25^, and South Korea^26^; smallpox virus in England^27^ and Europe^28^; and tuberculosis in Victoria, Australia^29^. To test if symptomatic status accurately predicts transmission potential, we also fit to a SARS-CoV-2 outbreak from Wanzhou, China in which the number of secondary cases have been separated into symptomatic and asymptomatic infectors across multiple generations^30^.

For each dataset, we first fit the negative binomial model to generate a canonical measure for the degree of overdispersion, $k_{\textrm{NB}}$. We then fit all compartmental model candidates, along with the canonical negative binomial model, and present visual comparisons of model fit and report their performance as measured by their maximum likelihood score, $\ell _{\textrm{max}}$, and corrected Akaike information criteria, AIC$_c$ (which accounts for small sample size, in addition to penalizing overparameterization). For each model, we also compare estimates of the reproductive number (*R*) and the probability of extinction (*q*). Finally, we analyze the estimated fraction of superspreaders among all infectious individuals (*c*), and the relative transmissibility of the sub- and superspreader classes ($\rho$) with reference to reported estimates where available.

## Results

### Transmission heterogeneity

**Figure 1:**
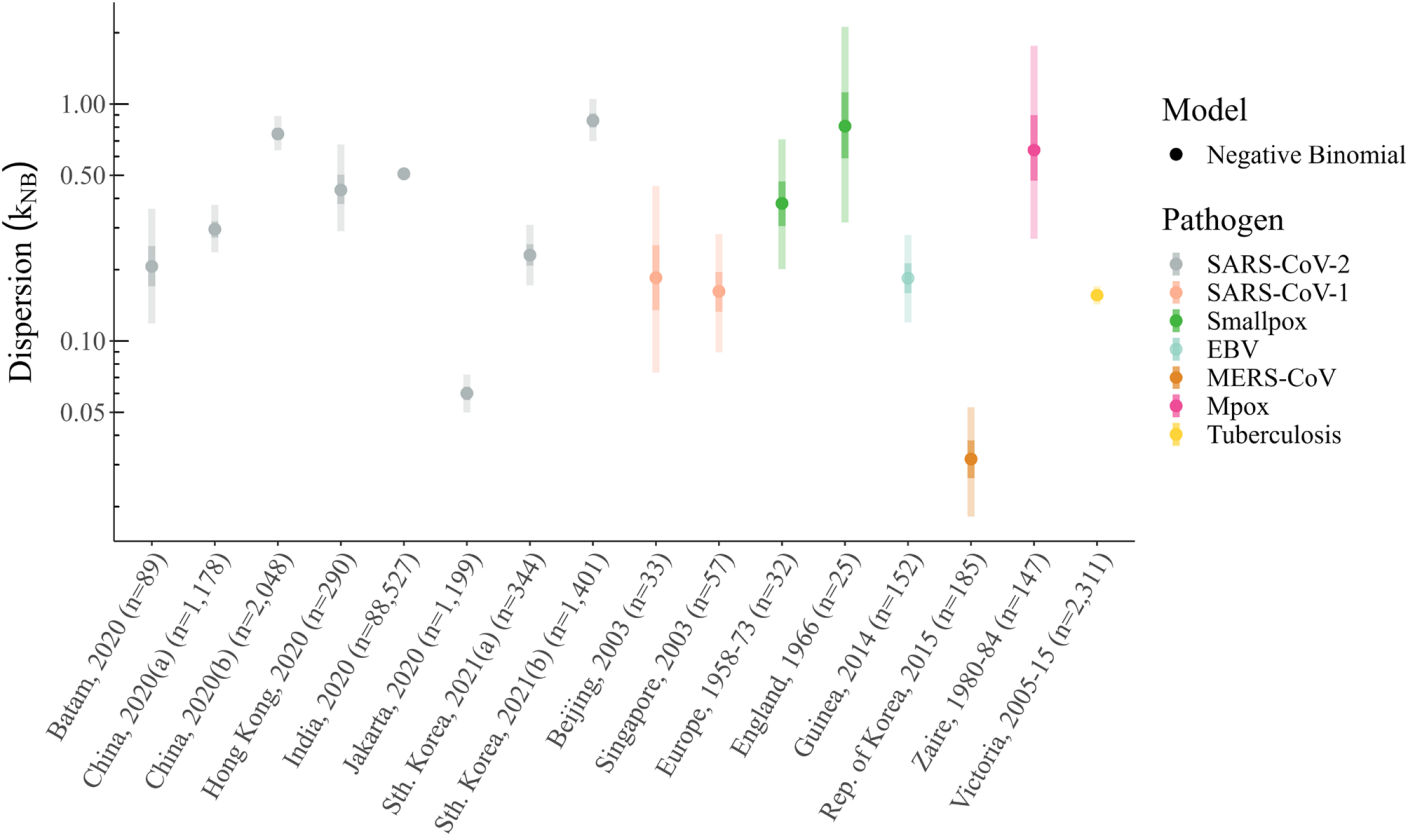
Estimates of the negative binomial dispersion parameter. Posterior estimates of the negative binomial dispersion parameter $k_{\textrm{NB}}$ for each of the 16 outbreak datasets included in our analysis. Markers indicate the median posterior estimate for $k_{\textrm{NB}}$ whilst the dark and light shaded bands give the 25–75% and 2.5–97.5% credible intervals, respectively. Each marker and interval is colored according to the corresponding pathogen: SARS-CoV-2 (gray); SARS-CoV-1 (salmon); smallpox (green); EBV (light blue); MERS-CoV (brown); Mpox (pink); and tuberculosis (yellow).

All 16 of the combined (i.e., those not split by clinical status) secondary case count datasets included in our analysis show evidence of considerable overdispersion, with median estimates of the negative binomial dispersion parameter $k_{\textrm{NB}}$ ranging from 0.03 (MERS-CoV) to 0.85 (SARS-CoV-2) (Fig. 1). Of the three pathogens with multiple datasets (i.e., SARS-CoV-2, SARS-CoV-1 and smallpox), only SARS-CoV-1 gives reasonably consistent dispersion estimates across each outbreak, with SARS-CoV-2 in particular exhibiting considerable variability (see Ref.^31^ for a recent review).

Similarly, for the Wanzhou, China dataset — where the offspring distribution is segregated according to symptomatic and asymptomatic infectors — we observe considerable overdispersion, with the 95% credible intervals all contained within $k_{\textrm{NB}} \le 1$. The lone exception is the first generation of symptomatic cases (prior to interventions) whose $k_{\textrm{NB}}$ 95% credible interval extends from 0.34 to 2.0.

### Model fits

**Figure 2:**
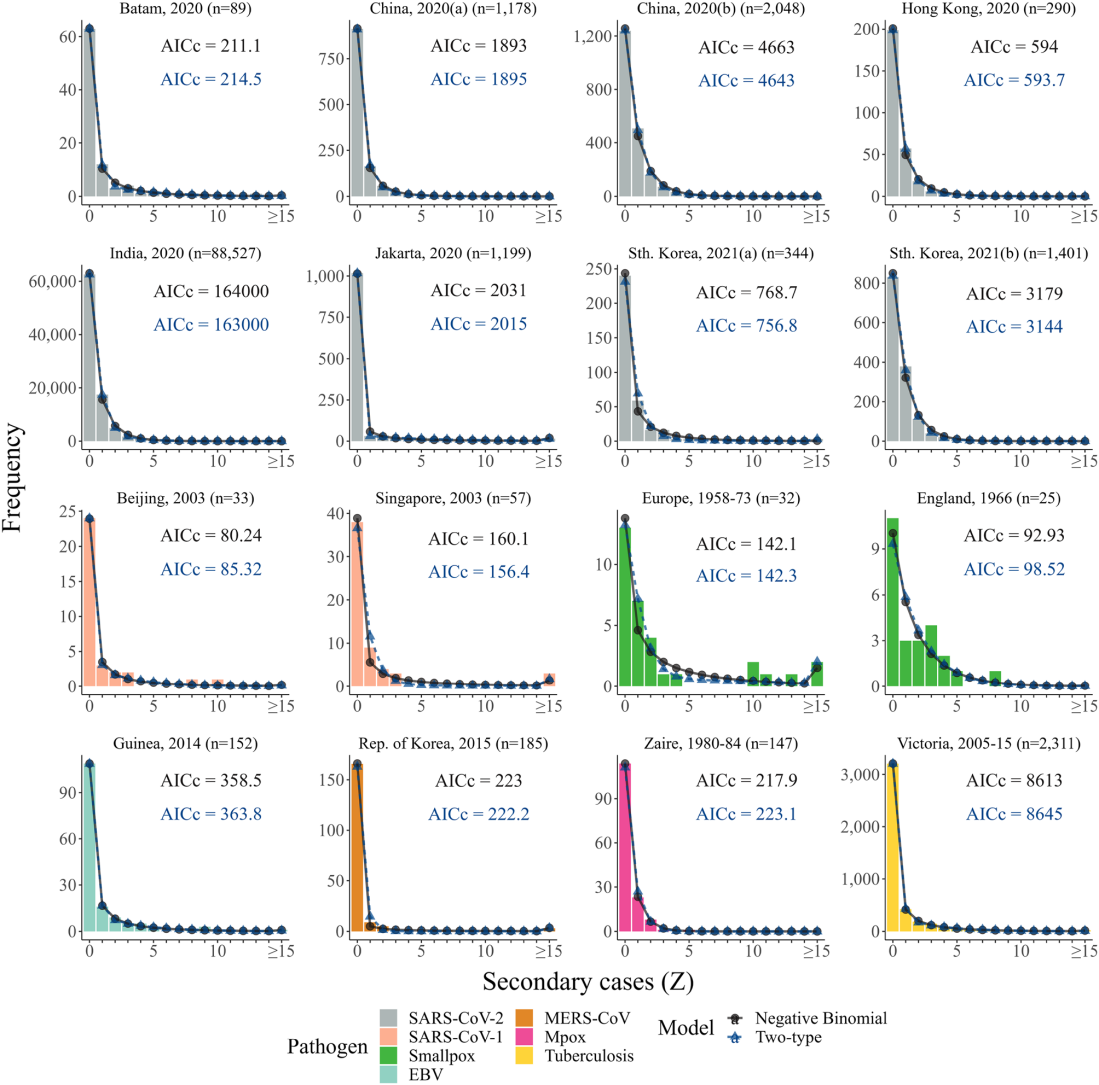
Model fits to secondary case counts. Secondary case count distributions for 16 outbreaks along with the best-fitting (according to maximum likelihood) negative binomial (black circles and solid line) and unconstrained two-type (blue triangle and dashed line) model predictions and their corresponding corrected Akaike information criteria (AIC$_c$). Each offspring distribution has been colored according to the corresponding pathogen: SARS-CoV-2 (gray); SARS-CoV-1 (salmon); smallpox (green); EBV (light blue); MERS-CoV (brown); Mpox (pink); and tuberculosis (yellow). Each panel is labelled by the location, year and size of each outbreak.

For the 16 combined secondary case count datasets considered, we found that the two-type compartmental model and its subvariants outperform (according to both maximum likelihood, $\ell _{\textrm{max}}$, and corrected Akaike information criteria, AIC$_c$) the benchmark negative binomial model eleven out of 16 times (Fig. 2 and Table S2). In four of the remaining five cases, the maximum likelihood values of the general, unconstrained two-type model are within one unit of the equivalent negative binomial score, and at least one of the two-type model or its subvariants has substantial or reasonable support (two with $\Delta$AIC$_c \le 2$, two with $2 \le \Delta$AIC$_c \le 4$). The 2005-15 Victorian tuberculosis dataset is the only offspring distribution for which all single- and two-type compartmental models are rejected.

Notably, within the two-type compartmental family, we found that SEIR-like variants (for which the serial relative transmissibility $\sigma =0$) possess the optimal AIC$_c$ value for 14 out of the 16 combined secondary case count datasets. The two exceptions are the SARS-CoV-2 outbreaks in India and South Korea(b), where the unconstrained ($\sigma \ne 0$) two-type model is preferred. Furthermore, we observed two occasions for which single-type models outcompeted the two-type candidates: the 1966 smallpox outbreak in the West Midlands region of England; and the 1984 Mpox outbreak in Zaire. In both cases, the SEIR-like variant was favoured (as measured by AIC$_c$).

The clinical model—where transmission potential is determined by symptomatic status—has either limited support (three out of 16) or is overwhelmingly rejected (13 out of 16) in all cases (Fig. S6).

**Figure 3:**
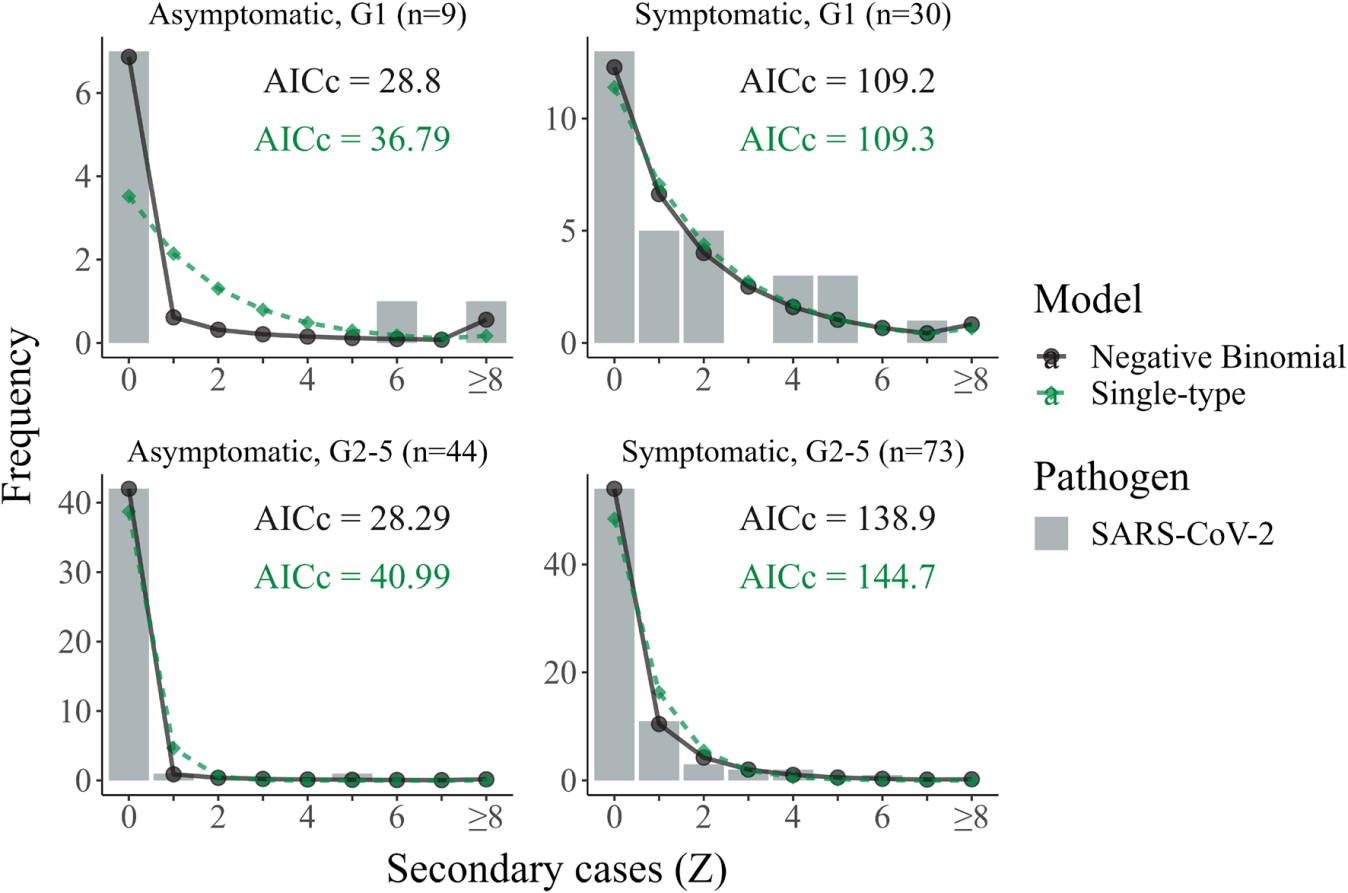
Comparison of model fits to clinically-segregated offspring distributions. Secondary case count distributions for the early 2020 SARS-CoV-2 outbreak in Wanzhou China across five generations of transmission, split by clinical status: asymptomatic—left panels; and symptomatic—right panels. The first generation (G1, prior to interventions) is shown in the upper row whilst generations two through to five are shown in the bottom row. Superimposed on each offspring distribution are the best-fitting (according to maximum likelihood) negative binomial (black circles and solid line) and single-type (green diamonds and dashed lined) model predictions and their corresponding corrected Akaike Information criteria (AIC$_c$).

For offspring data that are stratified by the symptomatic status of the infector, we find that the negative binomial model is typically favored (three out of the four distributions considered) and that the unconstrained single-type model (which is equivalent to the clinical model when applied to stratified data) is rejected in most cases (see Fig. 3).

### Parameter estimates

**Figure 4:**
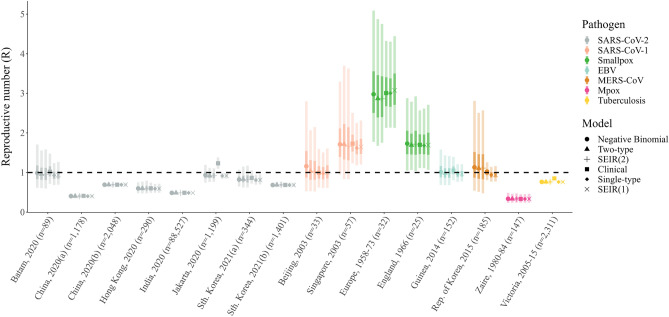
Reproductive number estimates. Model-specific posterior estimates of the reproductive number, *R*, for each of the 16 outbreak datasets included in our analysis. Markers indicate the median posterior estimate for *R*, for the negative binomial (circle), unconstrained two-type (triangle), two-type SEIR (plus), clinical (square), unconstrained single-type (diamond) and single-type SEIR (cross) models, whilst the dark and light shaded bands give the 25–75% and 2.5–97.5% credible intervals, respectively. Each marker and interval is colored according to the corresponding pathogen: SARS-CoV-2 (gray); SARS-CoV-1 (salmon); smallpox (green); EBV (light blue); MERS-CoV (brown); Mpox (pink); and tuberculosis (yellow). Each outbreak is labelled according to location, year and size. For reference, we also show the threshold value $R = 1$, indicated by the black dashed line.

Estimates of the reproductive number are mostly consistent across the different models considered, with little evidence of systematic bias (Fig. 4). Two notable exceptions are the 2020 SARS-CoV-2 outbreak in Jakarta, Indonesia and the 2005-15 tuberculosis surveillance data in Victoria, Australia, where the clinical model estimates a substantially higher reproductive number than each of the remaining models.

In general, single-type compartmental models provide more precise estimates of the reproductive number than either the two-type compartmental or negative binomial counterparts. Interestingly, median estimates of the reproductive number lie on alternate sides of the critical threshold $R=1$ for several datasets (e.g., SARS-CoV-2 in Jakarta and MERS-CoV in Korea), highlighting the potential impact of model selection on policy recommendations.

Analyzing the serial structure of each infectious type in the compartmental models considered, we found that the relative transmissibility of the first and second serial compartments, $\sigma$, was tightly constrained around zero for single-type models, whilst being considerably less constrained for two-type models (Fig. S7). The latter finding follows from the relatively flat likelihood curve as a function of this parameter in two-type models, where serial homogeneity seems to be compensated by parallel heterogeneity. Nevertheless, in most cases (across both single- and two-type variants) the maximum likelihood estimate for $\sigma$ was found to be approximately equal to zero — indicating that SEIR-like models were preferred (Table S3). This is consistent both with the model fitting results presented in the previous section, and our analytical analysis which confirmed that transmission heterogeneity is maximized when only a single serial infected compartment is actively infectious (see Supplement, Section 2).

**Figure 5:**
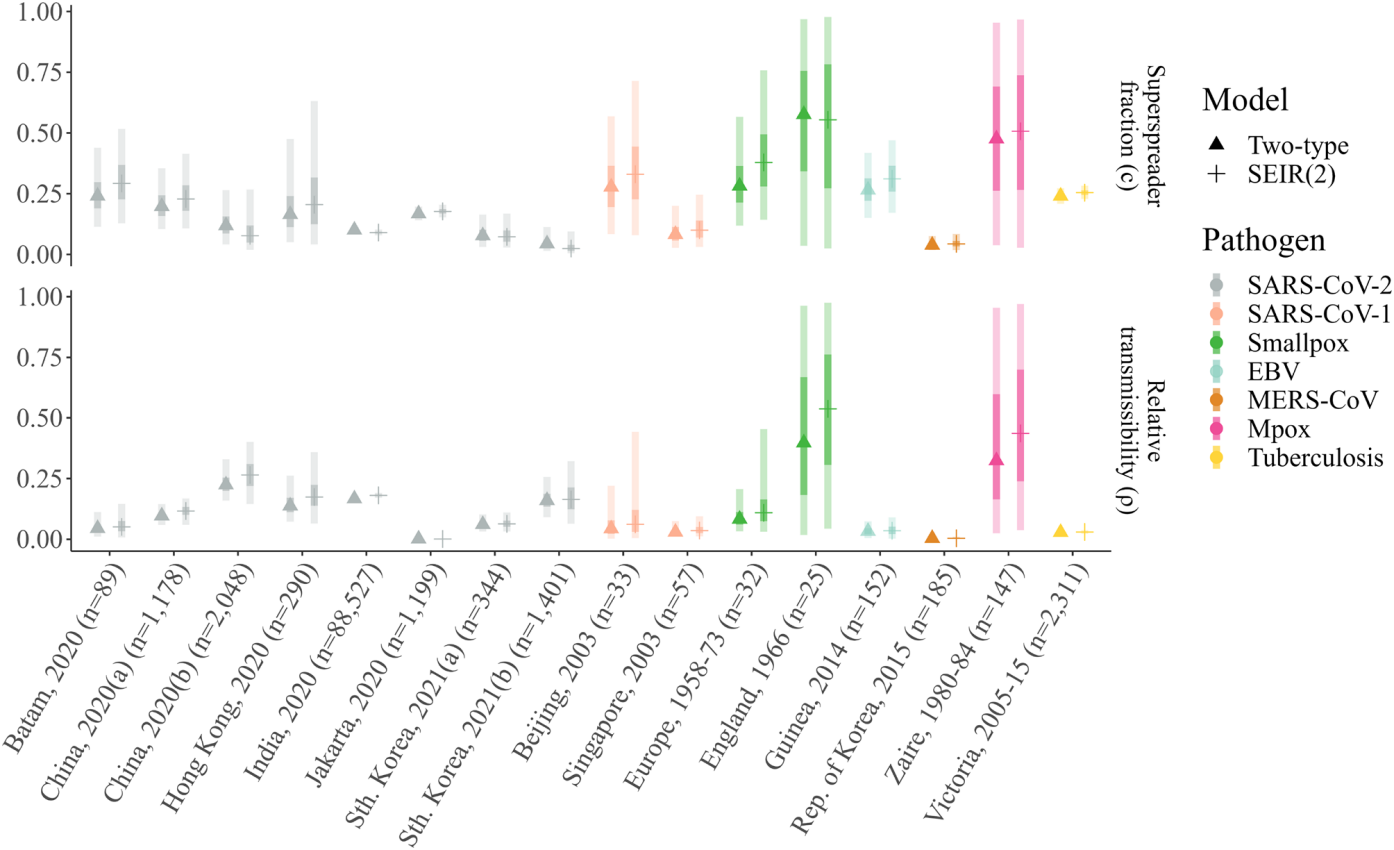
Estimated superspreader fraction and type-specific relative transmissibility. Posterior estimates of the (upper) superspreader fraction, *c*, and (lower) relative transmissibility, $\rho$, for each of the 16 outbreak datasets included in our analysis. Markers indicate the median posterior estimates of each parameter for the unconstrained two-type (triangle) and SEIR(2) (plus) models, whilst the dark and light shaded bands give the 25-75% and 2.5-97.5% credible intervals, respectively. Each marker and interval is colored according to the corresponding pathogen: SARS-CoV-2 (gray); SARS-CoV-1 (salmon); smallpox (green); EBV (light blue); MERS-CoV (brown); Mpox (pink); and tuberculosis (yellow). Each outbreak is labelled according to location, year and size.

Alternatively, for the parameters defining the type-specific structure of the model, we found the estimates of the superspreader fraction (*c*) and relative subspreader transmission potential $(\rho$) to be highly consistent across the two-type unconstrained ($\sigma \ne 0$) and SEIR ($\sigma =0$) models (Fig. 5). Moreover, for the subset of outbreaks for which two-type models are preferred over single-type alternatives (14 out of the 16 combined datasets considered) we find that the median estimated superspreader fraction (*c*) ranges from 3.8% for MERS-CoV to 37.9% for smallpox in Europe (Fig. 5, Table S3). Similarly, the median transmissibility of subspreaders relative to superspreaders ($\rho$) ranges from 0.1% for SARS-CoV-2 in Jakarta, Indonesia to 26.5% for SARS-CoV-2 in China(b). For the England smallpox and Zaire Mpox datasets (where the single-type SEIR-like model was preferred among the compartmental candidates), these parameters are relatively unconstrained. In most remaining cases, we find that the 95% credible interval for the superspreader fraction *c* lies well below and is non-overlapping with that of the observed clinical fraction of each pathogen (compare Fig. 5 with Table 2). For example, the credible intervals for *c* for the SARS-CoV-1 superspreader fraction range from 2.7% to 71.4%, which could be compared with an observed symptomatic fraction of 86.7% (95% CI 73.2–94.9%).

### Extinction probability

**Figure 6:**
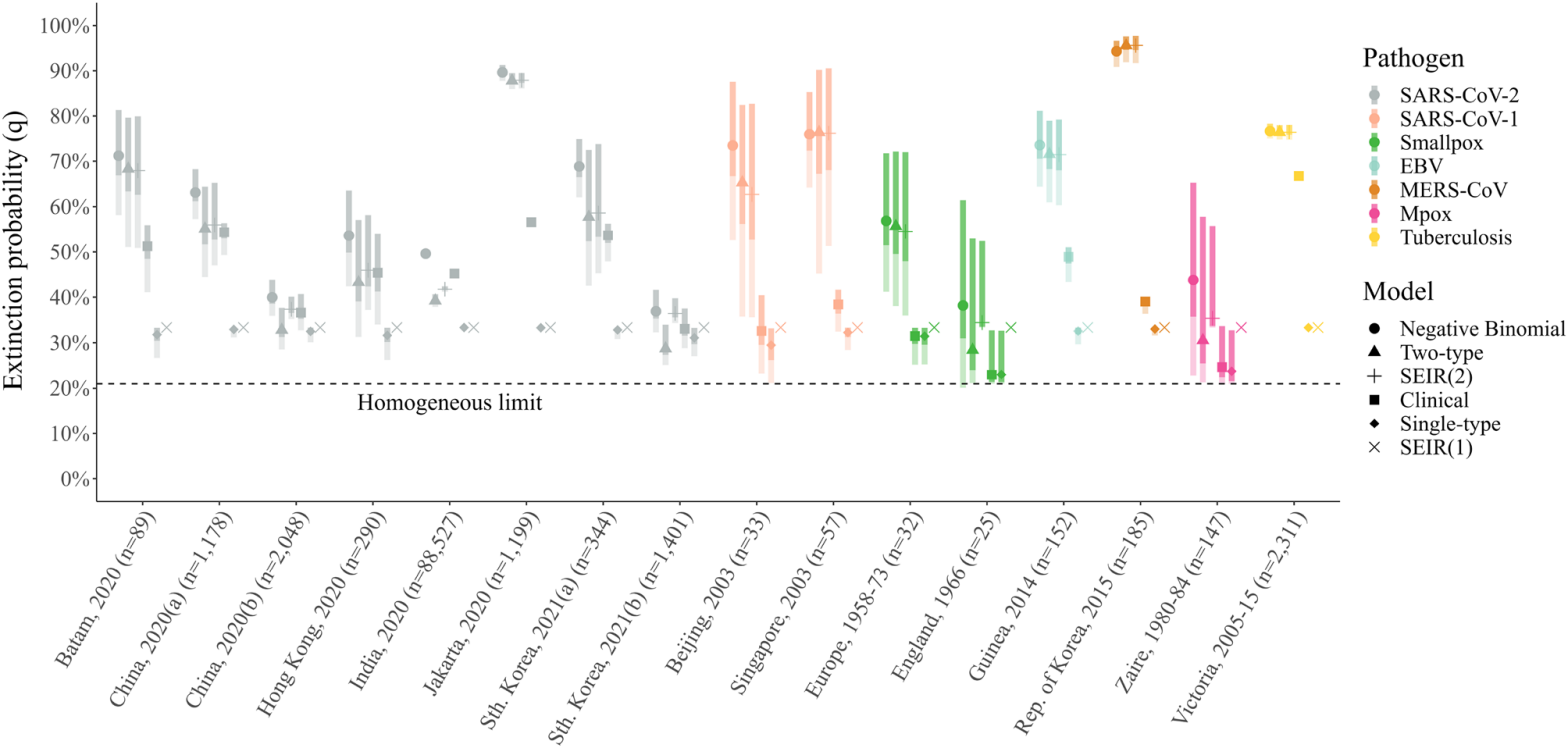
Extinction probability. Derived estimates of the extinction probability *q* using posterior estimates for the heterogeneity parameters $(\alpha , \sigma , c, \rho )$ and a fixed $R = 3$. Markers indicate the median extinction probability for the negative binomial (circle), unconstrained two-type (triangle), two-type SEIR (plus), clinical (square), unconstrained single-type (diamond) and single-type SEIR (cross) models, whilst the dark and light shaded bands give the 25-75% and 2.5-97.5% credible intervals, respectively. Each marker and interval is colored according to the corresponding pathogen: SARS-CoV-2 (gray); SARS-CoV-1 (salmon); smallpox (green); EBV (light blue); MERS-CoV (brown); Mpox (pink); and tuberculosis (yellow); and each outbreak is labelled according to location, year and size. For reference, we have also shown the predicted extinction probability for the single-type model with $\sigma =1$, i.e., $q\approx 0.209$.

The estimated probability of epidemic extinction is reasonably consistent across the negative binomial and two-type compartmental models (Fig. 6). Greater variability is observed for SARS-CoV-2 outbreaks, where the two-type model often predicts lower extinction probabilities. Exceptionally, the clinical model routinely predicts considerably lower extinction probabilities: a pattern that likely follows from its preponderance to underestimate the number of individuals with zero offspring (see Fig. S6). In all cases, the homogeneous model—in which all compartments have the same reproductive potential—provides a distinct lower bound on the probability of extinction, in agreement with the theoretical result that increasing heterogeneity promotes extinction^11^.

### Extended analysis

In our extended analysis, we investigated generalizations of the baseline two-type model by allowing for additional infectious types and greater numbers of serial compartments. We found that models with three parallel infectious streams (i.e., subspreaders, intermediate spreaders and superspreaders) provided excellent fits to the combined secondary case count data, producing the greatest maximum likelihood scores across all models (including the negative binomial benchmark) for 15 out of the 16 datasets considered. However, the additional parameters describing these models (an extra proportion and relative transmissiblity for the new type) meant that the penalized AIC$_c$ score was mostly sub-optimal. Nevertheless, three-type models were still favoured (by AIC$_c$) over single-, two-type and the benchmark negative binomial model for the SARS-CoV-2 outbreak in India and the tuberculosis surveillance data from Victoria. Similar to the two-type results above, we found that the performance of the three-type model was largely insensitive to the value of the serial relative transmissibility $\sigma$.

Conversely, increasing the number of actively infectious serial compartments within each type (which, for simplicity, we assumed all had the same reproductive potential), typically degraded the performance of the compartmental candidates (Fig. S8). However, in several instances there was either a decrease in AIC$_c$ as the number of actively infectious serial compartments increased (five out of 16 datasets), or the change was less than one unit (three out of 16).

Moreover, we found that incorporating additional infectious types successfully combated the homogeneity induced by lengthening the serial structure: a three-type SEIR-like model with five actively infectious compartments within each parallel infectious stream still produced better maximum likelihood scores than the benchmark negative binomial model for all 16 datasets, and was even preferred by AIC$_c$ for seven.

Finally, we found that changing the number of serial compartments had little effect on estimates of *R*, *c* and $\rho$.

## Discussion

Transmission heterogeneity strongly regulates the dynamics of burgeoning epidemics, presenting both challenges and opportunities for successful control^32^. In order to capitalize and guide outbreak responses, infectious disease models must capture this important epidemiological feature. In practice, compartmental models are often favored because they are comprehensible, flexible and tractable; however, the extent to which common models fully capture observed transmission heterogeneity remains unclear.

In this study we investigated the ability of compartmental models to replicate the extremely heterogeneous transmission patterns typical of infectious disease outbreaks. Using the canonical negative binomial branching process model as a benchmark, we found that compartmental models with at least two parallel infectious streams can capably reproduce observed superspreader dynamics. Within this class, we found that models with fewer actively infectious serial compartments (e.g., SEIR-like variants) generated greater heterogeneity, and that across the range of pathogens considered the optimal proportions of low-spreading individuals and their relative transmissibility ranged from 62.1–96.2%, and 0.1–26.5%, respectively.

The clinical model—where the proportion of individuals assigned to the sub- and superspreader classes is fixed by the observed symptomatic fraction of each pathogen—was strongly rejected for most outbreaks considered. This indicates that compartmental models stratified by a binary clinical classification routinely underestimate transmission heterogeneity, and miscalculate the epidemiological consequences that follow, e.g., the likelihood of epidemic extinction. Nonetheless, since symptomatic status is an identifiable characteristic, it is easier to parameterize models and interventions that are clinically-stratified^33^. Generating sufficient transmission heterogeneity then would require additional clinical sub-types that further segregate the symptomatic and asymptomatic sub-types (e.g., for TB we might consider smear-positive pulmonary, smear-negative pulmonary and extra-pulmonary cases). However, such extensions would also require more highly resolved clinical and surveillance data that detail the size and infectiousness of additional groups (e.g., the true superspreaders among those symptomatic).

Indeed, one limitation of our study was the decision to limit our analysis to two or fewer infectiousness types at baseline, and three or less in the extended analysis. This was motivated by wanting to find the simplest compartmental structure that would generate sufficient transmission heterogeneity, as determined by the negative binomial benchmark. Extending to multi-type models with more than three types would produce better fits and would be relatively straightforward to implement using the theoretical framework presented herein. Further, we note that other sources of individual variation such as differential susceptibility to infection and assortative mixing generate transmission heterogeneity, and it would be interesting to explore the extent to which these mechanisms reproduce observations.

Another limitation of our analysis is that we did not explicitly simulate or fit the temporal dynamics of infection, which is a primary reason for using the compartmental framework. A thorough investigation in which models are confronted with temporal data (e.g., incidence time series) would require bespoke structures for each pathogen (with e.g., incubation, pre-symptomatic transmission), which is beyond the scope of the present study. Instead, our objective was to fit observed offspring distributions, which depend only on the integrated reproductive potential $\nu$, and not the individual transmission and removal rates of each compartment. Nevertheless, the generalized structure considered herein permits wide-ranging temporal dynamics, which we demonstrated can be somewhat decoupled from integrated counts of secondary cases (see Supplement). This means that temporal homogeneity — if that is the goal — can be achieved without disturbing transmission heterogeneity. In any case, we showed that increasing the number of types can comfortably accommodate increases in temporal homogeneity, and that estimates of the transmissibility of each type and their relative proportion were reasonably robust to changes in model structure.

Ultimately, model design should be guided by the specific modelling objectives and the relevant data available, including known biological phenomena. Here we provide a reference of compartmental constructs and accompanying parameterizations that replicate observed transmission heterogeneity whilst maintaining temporal flexibility, allowing the latter to be constrained by alternative data sources.

Finally, we note that the results of this study also extend to other infectious disease modelling frameworks which have a direct compartmental model analogue (e.g., structured birth-death^34^ and coalescent^35^ phylodynamic models), and can be used to improve simulation and inference in these settings.

## Methods

### Offspring distributions

The probability distribution for the individual reproductive potential $\nu$ for the general model considered in our analysis is given by the following mixture density (for a detailed derivation of this, and all subsequent equations, see Supplement):

1 $$\begin{aligned} p(\nu ; \, {\textbf {R}}, \sigma , {\textbf {c}}) = \frac{1 + \sigma }{1 - \sigma }\sum _{i=1}^n \frac{c_i}{R_i}\textrm{e}^{-\nu /R_i}\left( \textrm{e}^{-\sigma \nu /R_i} - \textrm{e}^{-\nu /(\sigma R_i)} \right) , \end{aligned}$$

where $c_i$ is the proportion of the population assigned to each transmission type (e.g., subspreaders v. superspreaders), $R_{i}$ is the mean reproductive number of the *i*th type, and $\sigma$ is transmission potential of the first serial compartment relative to the second (which is assumed constant across types). At baseline, we only consider up to two transmission types (i.e., $i\in \{1,2\}$), however this is generalized to three in the extended analysis.

From this, we assume the number of secondary cases *Z* follows a Poisson distribution with rate parameter $\nu$, which yields

2 $$\begin{aligned} P(Z = z; {\textbf {R}}, \sigma , {\textbf {c}})&= \int _0^\infty P(Z=z|\nu )p(\nu ; {\textbf {R}}, \sigma , {\textbf {c}})\,d\nu ,\nonumber \\&= \frac{1 + \sigma }{1 - \sigma }\sum _{i=1}^n \frac{c_i}{R_i}\left[ \left( \frac{R_i}{1 + \sigma + R_i}\right) ^{z+1} - \left( \frac{\sigma R_i}{1 + \sigma + \sigma R_i}\right) ^{z+1} \right] . \end{aligned}$$

Rather than using the type-specific values, the two-type model has been parameterized in terms of: the population mean reproductive number, $R=c_1R_1 + c_2R_2$; the transmission potential of the first serial compartment within each type relative to the second $\sigma$; the proportion of the infected population that are superspreaders $c = c_2$ (which implies that $c_1 = 1 - c$); and the transmissibility of type 1 (subspreaders) relative to type 2 (superspreaders), $\rho = R_1 / R_2$. (Note that it follows from these definitions that $R\in [0,\infty )$, $\sigma \in [0,1]$, $c\in [0,1]$ and $\rho \in [0,1]$.) For the clinical sub-variant, the superspreader fraction is fixed by the observed symptomatic fraction for each pathogen (see Table 2). Alternatively, an SEIR-like sub-variant is obtained for $\sigma = 0$.

**Table 2:** Fraction of infections that are asymptomatic ($1-c_{\textrm{symp}}$).

Pathogen	Asymptomatic fraction* (95% CI)	Reference*
EBV	27.1% (14.5–39.6%)	^36^
	18.8% (12.3–27.3%)	^37^
MERS-CoV	10.2% (7.7–13.2%)	^38^
	12.1% (10.8–13.5%)	^39^
Mpox	4.8% (2.6–8.1%)	^40^
SARS-CoV-1	13.3% (5.1–26.8%)	^41^
SARS-CoV-2	35.1% (30.7–39.9%)	^42^
	30.8% (7.7–53.8%)	^43^
	40.5% (33.5–47.5%)	^44^
Smallpox	0%	^28^
Tuberculosis	50.4% (39.8–62.3%)$^\dagger$	^45^
	47.9% (45.0–50.8%)$^\ddagger$	^46^

*In the main analysis we use the central value of the first reported estimate for each pathogen.

$^\dagger$Inter-quartile range.

$^\ddagger$Smear negative proportion.

### Model fitting and derived parameters

Rather than explicitly simulating epidemic outbreaks as would be described by our general compartmental model, we use the predicted offspring distribution given in Eq. (2) to make direct comparisons with data. In this way we avoid the need to specify individual values for the transmission and removal rates of each compartment, and instead work directly with the integrated reproductive potential $\nu$ — which is a product of the transmission rate and infectious period of each compartment.

In particular, given a dataset *d* — which consists of a set of secondary case counts $\{Z_l^d\}_{l=1}^{N_d}$ for each individual *l* among the sampled set $N_d$—and a particular model *m* with parameters $\varvec{\theta }_m$, the likelihood is given by

$$\begin{aligned} L\left( \varvec{\theta }_m; \{Z_l^d\}_{l=1}^{N_d}\right)&= \prod _{l=1}^{N_d} P\left( Z = Z_l^d ; \varvec{\theta }_m\right) . \end{aligned}$$

For each dataset we estimated all model parameters $\varvec{\theta }_m$ using both maximum likelihood and Bayesian inference — the latter of which was specifically chosen to generate credible intervals for the probability of epidemic extinction. To facilitate comparison of parameter estimates across models, we used common prior distributions throughout: $R \sim \textrm{Gamma}(2,1)$ (all models); $k\sim \textrm{Exp}(1)$ (negative binomial model); $\sigma \sim U(0,1)$ (general and clinical models); $c \sim U(0,1)$ (two-type models); and $\rho \sim U(0,1)$ (two-type models).

To assess performance, we calculate the relative AIC$_c$ value for each model *m* and dataset *d*: $\Delta \textrm{AIC}_{c, m}^d = \textrm{AIC}_{c, m}^d - \textrm{AIC}_{c,\textrm{min}}^d$, and follow the guidelines described in Ref.^47^ by deeming that models with $\Delta \textrm{AIC}_{c, m} \le 2$ have substantial support, those with $4 \le \Delta \textrm{AIC}_{c, m} \le 7$ are not well supported, and those with $\Delta \textrm{AIC}_{c, m} > 10$ can be rejected. We also calculate the Akaike weight $w_m^d$ for each model, defined as

3 $$\begin{aligned} w_m^d = \frac{\textrm{e}^{-\frac{1}{2}\Delta \textrm{AIC}_{c, m}^d}}{\sum _m \textrm{e}^{-\frac{1}{2}\Delta \textrm{AIC}_{c, m}^d}}. \end{aligned}$$

For the Bayesian analyses we used a Hamiltonian Monte Carlo (HMC) sampler and ran five chains, assessing convergence and mixing by checking that all the parameters had effective sample sizes greater than 500 and that the $\hat{R}$ convergence diagnostic satisfied $|\hat{R} - 1| < 0.01$. The performance results are summarized in Table S2, and the parameter statistics are given in Table S3. Bayesian posterior distributions for $\sigma$, *c* and $\rho$ were then used to calculate posterior estimates for the probability of extinction (*q*) by solving the following transcendental equation for *q*:

4 $$\begin{aligned} q = \frac{1 + \sigma }{1 - \sigma } \sum _{i=1}^n c_i\left\{ \left[ 1 + \sigma + (1-q)R_i\right] ^{-1} - \sigma \left[ 1 + \sigma + (1-q) \sigma R_i\right] ^{-1} \right\}, \end{aligned}$$

where the quantity on the right is the generalized generating function for the mixture density (2). For the results shown in Fig. 6 we fixed the population mean $R = 3$ and used the posterior estimates for the remaining parameters.

### Supplementary Information

Supplementary Information.

## Data Availability

All code and date required to reproduce the results of this study can be found at https://github.com/AITHM/CompartmentalSuperspreaders.
