# Supplementary material for: Replicating superspreader dynamics with compartmental models
Source: Sci Rep. 2023 Sep 15;13:15319. doi: 10.1038/s41598-023-42567-3 (PMC10504364; doi:10.1038/s41598-023-42567-3)
Supplement: Supplementary file 1 — Supplementary Information. [file 41598_2023_42567_MOESM1_ESM.pdf]

Supplementary Materials

Michael T. Meehan, Angus Hughes, Romain R. Ragonnet,
Adeshina I. Adekunle, James M. Trauer, Pavithra Jayasundara,
Emma S. McBryde, Alec S. Henderson

August 11, 2023

1 Overview

In this work we investigate the capacity for compartmental models to replicate the transmission dynamics associated with observed outbreaks as represented by the distribution of secondary case counts for each primary infection. Throughout, we assume that the depletion of susceptibles as a result of infection is negligible (that is, we linearize our system about the infection-free equilibrium), allowing us to treat the number of secondary cases generated by each infectious individual as an independent, and identically distributed random variable, Z. This assumption also allows us to integrate over each individual's infectious period and treat transmission as a simple branching process. In this case the relevant quantity is the individual reproductive potential ν, which is the product of an individual's transmissibility per unit time (β) and their infectious period (T), i.e., $\nu = \beta T$. In this framework the parameter ν is the rate parameter in a Poisson process generating offspring Z. Given ν is itself a random variable with probability distribution $p(\nu; \boldsymbol{\theta}_m)$, the distribution for Z is calculated as

$$P(Z = z; \boldsymbol{\theta}_m) = \int_0^\infty P(Z = z|\nu)p(\nu; \boldsymbol{\theta}_m)\, d\nu \tag{1}$$

where the variable $\boldsymbol{\theta}_m$ is a vector of parameters specific to each compartmental model m.

In practice, this construction obviates the need to directly simulate the temporal dynamics of infection and allows us to immediately calculate the outcome measure of interest: the predicted offspring distribution $P(Z = z; \boldsymbol{\theta}_m)$. Importantly, this distribution is completely determined by the random variable ν (and the Poisson transmission model given ν), and is independent of the individual values of β and T — only their product. Nevertheless, in the sections that follow we also discuss the temporal dynamics generated by compartmental models illustrating how the candidate models we consider can generate wide-ranging temporal behaviour.

2 Dynamics

In this section we describe how the infection period and general reproductive potential distributions are derived from compartmental models, and suggest how temporal homogeneity and transmission heterogeneity might be achieved within a single modelling framework. For consistency of notation with the main article, in what follows we use the variable $i \in \{1, \dots, n\}$ to index the different infectious types, and the variable $j \in \{1, \dots, k\}$ to index the different serial compartments within each type.

2.1 SIR model

To begin, consider a simple SIR model with a single infectious compartment (Figure 1). Assuming that the recovery / removal rate δ from the infective compartment is a constant, we know that the infection period T is an exponentially distributed random variable with rate parameter δ:

$$T \sim \text{Exp}(\delta) \quad \rightarrow \quad p(T;\, \delta) = \delta e^{-\delta T}, \tag{2}$$

whose mean and variance are given respectively by

$$\mathbb{E}[T] = \frac{1}{\delta},$$
$$\text{var}[T] = \frac{1}{\delta^2}. \tag{3}$$

Further, assume that the transmission rate β is constant in time and across all individuals (i.e.,

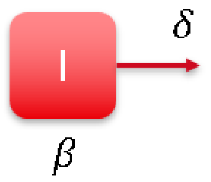

Figure 1: **Single infective compartment**. Single infective compartment with a constant transmission rate β and a constant removal / recovery rate δ.

not random), and that over the time period of interest the depletion of susceptibles is negligible. In this case the expected number of transmission events generated by each infected individual, which we will refer to as the reproductive potential ν, is calculated as

$$\nu = \beta T. \tag{4}$$

Using the properties for the function of a random variable (i.e., $\nu \equiv \nu(T)$) we can straightforwardly derive the probability distribution for ν using (2):[1]

$$p(\nu; \delta, \beta) = \frac{\delta}{\beta} \mathrm{e}^{-\delta\nu/\beta}. \tag{6}$$

That is, an exponentially distributed infectious period T yields an exponentially-distributed reproductive potential ν. Once again, the mean and variance of this distribution are given by

$$\begin{aligned} \mathbb{E}[\nu] &= \frac{\beta}{\delta} \equiv R, \\ \mathrm{var}[\nu] &= \left(\frac{\beta}{\delta}\right)^2 = \frac{1}{R^2} \end{aligned} \tag{7}$$

where we have defined the reproductive number $R = \mathbb{E}[\nu] = \beta\mathbb{E}[T]$ as the mean of the reproductive potential distribution.

Note that in the distribution for the reproductive potential (6), the transmission rate β and recovery rate δ always appear as the ratio β/δ. Therefore, the parameter ν is entirely determined by the reproductive number R, allowing us to rewrite (6) as

$$p(\nu; R) = \frac{1}{R} \mathrm{e}^{-\nu/R}. \tag{8}$$

This means that when working with the reproductive potential ν and the consequent number of secondary cases, we do not need to specify the individual rates of transmission and removal, only their ratio, R.

2.2 Serial models

Building on the formalism from the previous section, we now consider extended SIR-like models in which an arbitrary number (k) of infectious compartments are placed in series (Figure 2). Upon infection individuals enter the first compartment I_1 and transition through the remaining $j \in \{2, \ldots, k\}$ serial infective compartments sequentially. Importantly, the time spent in each compartment is an independent (not necessarily identically-distributed) random variable T_j, such that the total transit time $T = \sum_{j=1}^{k} T_j$ follows a HypoExponential distribution:

$$T \sim \mathrm{HypoExp}(\delta_1, \ldots, \delta_k) \tag{9}$$

where δ_j is the removal rate from the jth infective compartment. As a specific example, with only

[1] The probability distribution for ν can be derived by using the identity for the probability distribution of a function of a random variable, $Y = g(X)$:

$$f_Y(y) = f_X(g^{-1}(y)) \left| \frac{dg^{-1}(y)}{dy} \right|. \tag{5}$$

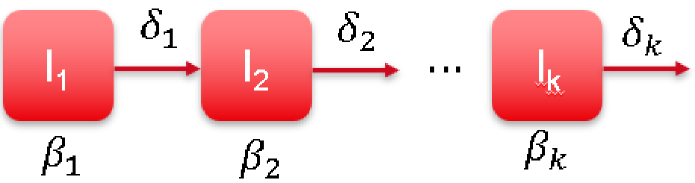

Figure 2: **Serial compartmental model**. Compartmental model composed of a series of infective compartments, each with unique transmission rates β_j and removal rates δ_j.

two serial compartments with differing removal rates $\delta_1 \neq \delta_2$, the infectious period distribution is given by

$$T \sim \text{HypoExp}\left(\delta_1, \delta_2\right) \quad \rightarrow \quad p(T;\, \delta_1, \delta_2) = \frac{\mathrm{e}^{-\delta_1 T} - \mathrm{e}^{-\delta_2 T}}{\delta_2^{-1} - \delta_1^{-1}} \qquad \delta_1 \neq \delta_2. \tag{10}$$

Alternatively, for a model with k infective compartments, each with equal removal rates δ, we recover the Erlang distribution:

$$T \sim \text{Erlang}(\delta, k) \qquad \rightarrow \qquad p(T; \delta, k) = \frac{\delta^k x^{k-1} \mathrm{e}^{-\delta T}}{(k-1)!}. \tag{11}$$

In direct analogy with the single-infective-compartment case (i.e., the simple SIR model), we find that the probability distribution for the total reproductive potential $\nu = \sum_{j=1}^{k} \nu_j$ also generalizes to a HypoExponential distribution in the serial case:

$$\nu \sim \text{HypoExp}(R_1^{-1}, \ldots) \tag{12}$$

where $R_j = \beta_j/\delta_j$ are the mean reproductive numbers for each of the individual compartments. **Note:** In this parameterization it is understood that each of the included R_j are non-zero. This does not preclude the addition of non-infectious compartments (as in e.g., the SEIR model), only that such compartments will not modify the reproductive potential distribution. This is a useful property that can be exploited to adjust the temporal dynamics of infection, whilst leaving the offspring component unaltered.

In general, the mean and variance of a HypoExponentially-distributed random variable $X \sim \text{HypoExp}(\lambda_1, \ldots, \lambda_k)$ are given respectively by

$$\begin{aligned} \mathbb{E}[X] &= \sum_{j=1}^{k} \mathbb{E}[X_j] = \sum_{j=1}^{k} \frac{1}{\lambda_j}, \\ \text{var}[X] &= \sum_{j=1}^{k} \text{var}[X_j] = \sum_{j=1}^{k} \frac{1}{\lambda_j^2}. \end{aligned} \tag{13}$$

That is, for serial compartmental models, the total mean is just the sum of the individual compartmental means, and likewise for the variance. (This result follows directly from the independence of the T_js and ν_js.)

Before proceeding, we highlight that the total variance given in the formula above can be bounded both above and below:

$$\frac{1}{k}\left(\sum_{j=1}^{k} \frac{1}{\lambda_j}\right)^2 \leq \sum_{j=1}^{k} \frac{1}{\lambda_j^2} \leq \left(\sum_{j=1}^{k} \frac{1}{\lambda_j}\right)^2. \tag{14}$$

In terms of the mean and variance this becomes

$$\frac{\left(\mathbb{E}[X]\right)^2}{k} \leq \text{var}[X] \leq \left(\mathbb{E}[X]\right)^2. \tag{15}$$

Therefore, for a fixed a mean, the variance is minimized when λ_j is constant for all j; and it is maximized when only a single $\lambda_j \neq 0$ and the summation collapses.

Importantly, if we refer back to our serial compartmental model, we then see that **temporal variance is minimized when each of the δ_j are equal, whilst transmission variance is maximized when only a single $\beta_j \neq 0$.** This circumstance can be realized in SEIR-like models, where a single infective compartment, I, is surrounded by non-infectious compartments, E, and all compartments have a common removal rate (Figure 3).

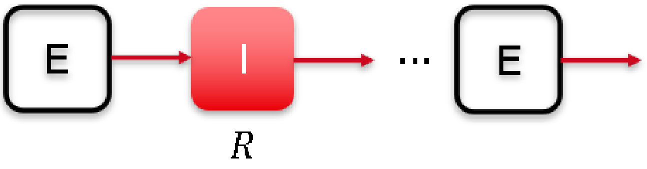

Figure 3: **SEIR-like compartmental model**. Serial compartmental model in which only one of the compartments is actively infectious, with mean reproductive number R.

2.3 Multi-type models

The analysis above can be generalized further by considering multi-type models where the chains of serial compartments are structured into parallel infectious streams (Figure 4). Assuming that

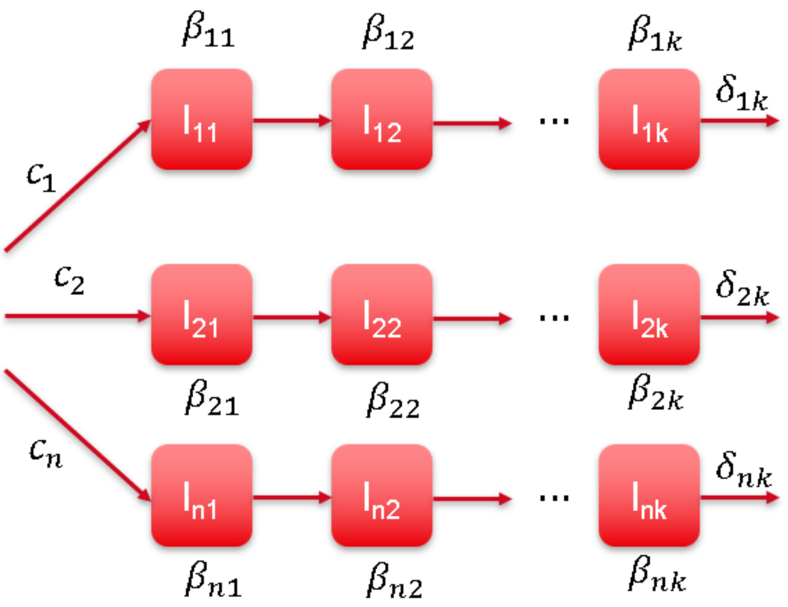

Figure 4: **Multi-type compartmental model**. Compartmental model composed of n parallel infectious streams, with each stream consisting of k serial compartments. The proportion of individuals assigned to the ith stream is denoted c_i, and the recovery and removal rates of the ijth compartment are β_{ij} and δ_{ij}, respectively.

individuals are assigned to a particular infectious stream i with probability c_i, the probability densities for the infection period T and the reproductive potential ν take the general form of a mixture density:

$$p(X;\theta) = \sum_{i=1}^{n} c_i p_i(X_i;\theta_i) \tag{16}$$

where X is a placeholder for either T or ν, X_i is the type-specific value of either parameter, and θ_i is a vector of rate parameters characterizing the type-specific distributions p_i. As noted above, the serial structure of each infectious stream results in a HypoExponential distribution for both T_i and ν_i, with general rate parameters λ_{ij}.

The mean of the mixture distribution (16) is calculated as the weighted sum of the type-specific means:

$$\mathbb{E}[X] = \sum_{i=1}^{n} c_i \mathbb{E}[X_i], \tag{17}$$

whilst the variance is less straightforward:

$$\mathrm{var}[X] = \sum_{i=1}^{n} c_i \left[\mathrm{var}[X_i] + (\mathbb{E}[X_i])^2\right] - \left(\sum_{i=1}^{n} c_i \mathbb{E}[X_i]\right)^2. \tag{18}$$

From this relation we see that higher order moments of mixture distributions can display exotic behaviour, with e.g., variances that can potentially exceed that of any of their individual components.

In this way we can use mixtures of HypoExponential distributions with varying reproductive potentials across each type to generate arbitrarily heterogeneous transmission dynamics. Moreover, if the removal rates are held fixed across each type (i.e., $\delta_{ij} = \delta_{i'j}$), we can still preserve any temporal homogeneity originally achieved by placing the compartments in series. (In this latter case, the $p_i(T_i)$ are equal and the mixture distribution for the infectious period collapses back to a standard HypoExponential model.)

3 Candidate model

In this study we propose a multi-type compartmental model that we hope can capably reproduce homogeneous temporal dynamics and heterogeneous transmission dynamics. Motivated by the results of the previous section, we propose a baseline model that is composed of two parallel infectious streams (a sub-spreader type and a superspreader type), with each stream consisting of two serial compartments. The proportion of individuals assigned to the superspreader type is c (see Figure 5).

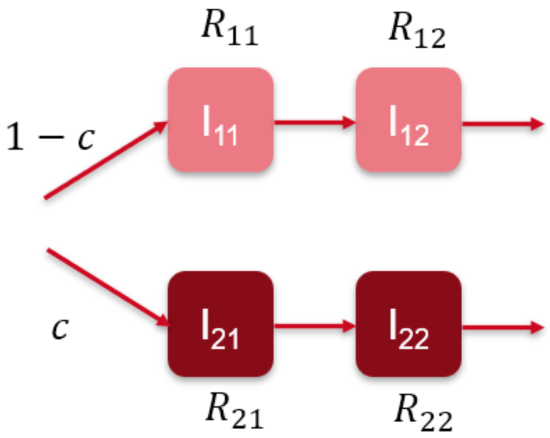

Figure 5: **Two-type baseline model**. Baseline compartmental model used in our main analysis composed of a superspreader type (dark red) and a subspreader type (pink), with each type consisting of two serial compartments. The proportion of individuals assigned to the superspreader type is c. The recovery and removal rates of the ijth compartment are β_{ij} and δ_{ij}, respectively.

Using the results presented in the previous sections for the mean of HypoExponential and mixture distributions, it immediately follows that the mean type-specific reproductive numbers, R_i, and total reproductive number, R, for this model are given respectively by

$$R_i = R_{i1} + R_{i2} \tag{19}$$

and

$$R = (1 - c)R_1 + cR_2. \tag{20}$$

If we use the variable $\rho = R_1/R_2$ to define the transmission potential of subspreaders (type 1) relative to superspreaders (type 2), we can invert the equation above to specify the type-specific reproductive numbers in terms of the total reproductive number:

$$\begin{aligned} R_1 &= \frac{\rho R}{c + \rho(1 - c)}, \\ R_2 &= \frac{R}{c + \rho(1 - c)}. \end{aligned} \tag{21}$$

Moreover, if we introduce the variable $\sigma = R_{i1}/R_{i2}$ to denote transmission potential of the first serial compartment relative to that of the second, we obtain

$$\begin{aligned} R_{i1} &= \frac{\sigma}{1 + \sigma} R_i, \\ R_{i2} &= \frac{1}{1 + \sigma} R_i. \end{aligned} \tag{22}$$

For simplicity, we assume that the ratio σ is constant across types, and without loss of generality we assume that $R_1 \le R_2$ such that $0 \le \sigma \le 1$.

Given the above, we choose to parameterize our model in terms of:

- R — the mean reproductive potential of the entire compartmental network;
- σ — the ratio of the reproductive number of the first serial compartment relative to the second: R_{i1}/R_{i2};
- c — the proportion of infected individuals assigned to the superspreader type; and
- ρ — the ratio of the reproductive number of subspreaders relative to superspreaders: R_1/R_2.

3.1 Reproductive potential

The probability distribution for the total reproductive potential ν for the baseline compartmental model introduced above is a mixture of HypoExponential distributions, namely:

$$p(\nu; R_{11}, R_{12}, R_{21}, R_{22}, c) = (1-c)\frac{\mathrm{e}^{-\nu/\mathrm{R}_{11}} - \mathrm{e}^{-\nu/R_{12}}}{R_{12} - R_{11}} + c\frac{\mathrm{e}^{-\nu/\mathrm{R}_{21}} - \mathrm{e}^{-\nu/R_{22}}}{R_{22} - R_{21}}. \tag{23}$$

In terms of the relative transmission potential σ and the type-specific reproductive potentials R_i, this becomes

$$p(\nu; R_1, R_2, \sigma, c) = \frac{1+\sigma}{1-\sigma}\left[c\frac{\mathrm{e}^{-\nu/R_1}}{R_1}\left(\mathrm{e}^{-\sigma\nu/R_1} - \mathrm{e}^{-\nu/(\sigma R_1)}\right) + (1-c)\frac{\mathrm{e}^{-\nu/R_2}}{R_2}\left(\mathrm{e}^{-\sigma\nu/R_2} - \mathrm{e}^{-\nu/(\sigma R_2)}\right)\right]. \tag{24}$$

Note that here we have defined the distribution in terms of R_1 and R_2 for ease of notation only. In practice, we use the alternative parameterization R and ρ.

3.2 Offspring distribution

Modelling transmission as a Poisson point process with rate parameter ν, the number of secondary cases Z generated by each primary infection throughout their infectious lifetime is given by

$$P(Z = z|\nu) = \frac{\nu^z \mathrm{e}^{-\nu}}{z!}. \tag{25}$$

Using the mixture distribution (24) to integrate over the reproductive potential ν yields:

$$\begin{aligned} P(Z = z; R_1, R_2, \sigma, c) &= \int_0^\infty P(Z = z|\nu) p(\nu; R_1, R_2, \sigma, c)\, d\nu, \\ &= \frac{1+\sigma}{1-\sigma}\sum_{i=1}^{n} c_i \frac{1}{R_i}\left[\left(\frac{R_i}{1+\sigma+R_i}\right)^{z+1} - \left(\frac{\sigma R_i}{1+\sigma+\sigma R_i}\right)^{z+1}\right] \end{aligned} \tag{26}$$

where in the two-type baseline model $n = 2$, $c_2 = c$ and $c_1 = 1 - c$.

This result can be compared with the analogous expression used for the canonical Negative Binomial model (where the reproductive potential $\nu \sim \Gamma(R, k)$):

$$P(Z = z; R, k) = \binom{z+k-1}{z}\left(\frac{k}{k+R}\right)^k \left(\frac{R}{k+R}\right)^z. \tag{27}$$

3.3 Special cases

The baseline model introduced at the start of this section admits a number of special cases for limiting values of the parameters σ, c and ρ. We especially note the following:

- SEIR(2) ($\sigma = 0$): In the limit $\sigma \to 0$ the first serial compartment of each type becomes non-transmissible and the general baseline model reduces to a two-type SEIR model.
- Clinical ($c = c_{\mathrm{symp}}$): We can fix the fraction of the individuals entering the superspreader class according to the fraction of symptomatic individuals c_{symp} to recover the clinical-type model.
- Single-type ($c = 0$): In this case all individuals are assigned to type 1.
- SEIR(1) ($c = \sigma = 0$): Here we consider only a single type where the first serial compartment is non-transmissible.

The offspring distributions for each of these reduced models can be derived directly from the general case, equation (24).

4 Extended analysis

As part of our extended analysis, we generalize the baseline model to include larger numbers of serial infective compartments $k = 2, 3$. To avoid the introduction of numerous new parameters, and to keep the model from becoming unwieldy, we assume that the transmission potential of all active serial infectious compartments is the same. As above, we note that the arbitrary inclusion of non-transmissible compartments is still permitted, and the results are immediately generalizable to this case. As such, the variable k merely counts the serial number of transmissible states.

To generate increased heterogeneity we also considered models with three parallel infectious streams, i.e., three-type models. In particular, we consider a three-type extension of the baseline two-type model with two serial compartments and where differential transmissibility is permitted across each serial compartment (the unconstrained three-type model), and we also consider a three-type SEIR-like model in which the first serial compartment in each of the three types is non-transmissible. This latter model is also extended to an arbitrary number of equally transmissible serial infective states.

Type	Model	Fixed parameters	Free parameters	(#)
Three-type	General(3) (unconstrained)	—	R, σ, c_1, c_2, ρ_1, ρ_2	(6)
	SEIR(3)	$\sigma = 0$	R, c_1, c_2, ρ_1, ρ_2	(5)

Table 1: Three-type model parameter summary.

5 Model fitting

Having derived general expressions for the offspring distributions of our candidate models, we then proceed to parameter estimation using observed secondary case count distributions from the 16 outbreaks listed in the main article. Each dataset d consists of a set of secondary case counts $\{Z_l^d\}_{l=1}^{N_d}$ for each individual l among the sampled set N_d. Given a particular model m with parameters $\boldsymbol{\theta}_m$, the likelihood of observing the secondary case count distribution $\{Z_l^d\}_{l=1}^{N_d}$ from dataset d is

$$L\left(\boldsymbol{\theta}_m; \{Z_l^d\}_{l=1}^{N_d}\right) = \prod_{l=1}^{N_d} P\left(Z = Z_l^d; \boldsymbol{\theta}_m\right). \tag{28}$$

For the unconstrained, two-type baseline model, this becomes

$$L(R_1, R_2, c, \sigma; \{Z_l^d\}_{l=1}^{N_d}) = \left(\frac{1+\sigma}{1-\sigma}\right)^{N_d} \prod_{i=1}^{N_d} \left\{ \sum_{i=1}^{2} c_i \frac{1}{R_i} \left[\left(\frac{R_i}{1+\sigma+R_i}\right)^{Z_l^d+1} - \left(\frac{\sigma R_i}{1+\sigma+\sigma R_i}\right)^{Z_l^d+1} \right] \right\}. \tag{29}$$

For the Negative Binomial model, which we use as a benchmark, we alternatively have

$$L(R, k; \{Z_l^d\}_{l=1}^{N_d}) = \left(\frac{k}{k+R}\right)^{kN_d} \left(\frac{R}{k+R}\right)^{\sum_{i=1}^{N_d} Z_l^d} \prod_{i=1}^{N_d} \binom{Z_l^d + k - 1}{Z_l^d}. \tag{30}$$

The maximum likelihood estimate $\hat{\boldsymbol{\theta}}_m^d$ for each dataset d and model m is determined by maximizing the likelihood function $L\left(\boldsymbol{\theta}_m; \{Z_l^d\}_{l=1}^{N_d}\right)$ over the parameter vector space Θ_m:

$$\hat{\boldsymbol{\theta}}_m^d = \underset{\boldsymbol{\theta}_m \in \Theta_m}{\text{argmax}}\, L\left(\boldsymbol{\theta}_m; \{Z_l^d\}_{l=1}^{N_d}\right). \tag{31}$$

Details of the Bayesian model fitting, including the choice of priors for each parameter can be found in the Methods section of the main article.

Pathogen	Location	n_{obs}	Model	$\ell_{\max}$	BIC	AIC	AIC$_c$	w
SARS-CoV-2	Batam	89	NB	-1.035×10^2	2.159×10^2	2.109×10^2	2.111×10^2	0.49
			TT	-1.030×10^2	2.239×10^2	2.140×10^2	2.145×10^2	0.09
			Clinical	-1.075×10^2	2.285×10^2	2.210×10^2	2.213×10^2	0.00
			SEIR(2)	-1.031×10^2	2.196×10^2	2.121×10^2	2.124×10^2	0.25
			ST	-1.176×10^2	2.443×10^2	2.393×10^2	2.394×10^2	0.00
			SEIR(1)	-1.176×10^2	2.398×10^2	2.373×10^2	2.373×10^2	0.00
	China	1178	NB	-9.444×10^2	1.903×10^3	1.893×10^3	1.893×10^3	0.49
			TT	-9.437×10^2	1.916×10^3	1.895×10^3	1.895×10^3	0.14
			Clinical	-9.539×10^2	1.929×10^3	1.914×10^3	1.914×10^3	0.00
			SEIR(2)	-9.437×10^2	1.909×10^3	1.893×10^3	1.893×10^3	0.37
			ST	-9.852×10^2	1.984×10^3	1.974×10^3	1.974×10^3	0.00
			SEIR(1)	-9.852×10^2	1.977×10^3	1.972×10^3	1.972×10^3	0.00
		2048	NB	-2.329×10^3	4.674×10^3	4.663×10^3	4.663×10^3	0.00
			TT	-2.317×10^3	4.665×10^3	4.643×10^3	4.643×10^3	0.50
			Clinical	-2.327×10^3	4.677×10^3	4.660×10^3	4.660×10^3	0.00
			SEIR(2)	-2.318×10^3	4.660×10^3	4.643×10^3	4.643×10^3	0.50
			ST	-2.335×10^3	4.685×10^3	4.674×10^3	4.674×10^3	0.00
			SEIR(1)	-2.335×10^3	4.678×10^3	4.672×10^3	4.672×10^3	0.00
	Hong Kong	290	NB	-2.950×10^2	6.013×10^2	5.939×10^2	5.940×10^2	0.18
			TT	-2.928×10^2	6.082×10^2	5.936×10^2	5.937×10^2	0.21
			Clinical	-2.963×10^2	6.097×10^2	5.987×10^2	5.988×10^2	0.02
			SEIR(2)	-2.928×10^2	6.026×10^2	5.916×10^2	5.916×10^2	0.58
			ST	-3.020×10^2	6.154×10^2	6.080×10^2	6.081×10^2	0.00
			SEIR(1)	-3.020×10^2	6.097×10^2	6.060×10^2	6.060×10^2	0.00
	India	88527	NB	-8.199×10^4	1.640×10^5	1.640×10^5	1.640×10^5	0.00
			TT	-8.149×10^4	1.630×10^5	1.630×10^5	1.630×10^5	1.00
			Clinical	-8.212×10^4	1.643×10^5	1.642×10^5	1.642×10^5	0.00
			SEIR(2)	-8.150×10^4	1.630×10^5	1.630×10^5	1.630×10^5	0.00
			ST	-8.298×10^4	1.660×10^5	1.660×10^5	1.660×10^5	0.00
			SEIR(1)	-8.298×10^4	1.660×10^5	1.660×10^5	1.660×10^5	0.00
	Jakarta	1199	NB	-1.013×10^3	2.041×10^3	2.031×10^3	2.031×10^3	0.00
			TT	-1.003×10^3	2.035×10^3	2.015×10^3	2.015×10^3	0.09
			Clinical	-1.299×10^3	2.619×10^3	2.604×10^3	2.604×10^3	0.00
			SEIR(2)	-1.003×10^3	2.028×10^3	2.013×10^3	2.013×10^3	0.25
			ST	-1.588×10^3	3.191×10^3	3.181×10^3	3.181×10^3	0.00
			SEIR(1)	-1.588×10^3	3.184×10^3	3.179×10^3	3.179×10^3	0.00
	South Korea (a)	344	NB	-3.823×10^2	7.763×10^2	7.686×10^2	7.687×10^2	0.00
			TT	-3.743×10^2	7.720×10^2	7.566×10^2	7.568×10^2	0.26
			Clinical	-3.993×10^2	8.161×10^2	8.046×10^2	8.047×10^2	0.00
			SEIR(2)	-3.743×10^2	7.662×10^2	7.546×10^2	7.547×10^2	0.74
			ST	-4.268×10^2	8.653×10^2	8.576×10^2	8.577×10^2	0.00
			SEIR(1)	-4.268×10^2	8.595×10^2	8.556×10^2	8.557×10^2	0.00
	South Korea (b)	1401	NB	-1.588×10^3	3.190×10^3	3.179×10^3	3.179×10^3	0.00
			TT	-1.568×10^3	3.165×10^3	3.144×10^3	3.144×10^3	0.94
			Clinical	-1.584×10^3	3.190×10^3	3.174×10^3	3.174×10^3	0.00
			SEIR(2)	-1.572×10^3	3.165×10^3	3.149×10^3	3.149×10^3	0.06
			ST	-1.589×10^3	3.192×10^3	3.181×10^3	3.181×10^3	0.00
			SEIR(1)	-1.589×10^3	3.185×10^3	3.180×10^3	3.180×10^3	0.00

Pathogen	Location	n_{obs}	Model	$\ell_{\max}$	BIC	AIC	AIC$_c$	w
EBV	Guinea	152	NB	-1.772×10^2	3.644×10^2	3.584×10^2	3.585×10^2	0.67
			TT	-1.777×10^2	3.756×10^2	3.635×10^2	3.638×10^2	0.05
			Clinical	-1.897×10^2	3.945×10^2	3.855×10^2	3.856×10^2	0.00
			SEIR(2)	-1.777×10^2	3.706×10^2	3.615×10^2	3.616×10^2	0.14
			ST	-2.058×10^2	4.216×10^2	4.156×10^2	4.156×10^2	0.00
			SEIR(1)	-2.058×10^2	4.166×10^2	4.136×10^2	4.136×10^2	0.00
MERS-CoV	Korea	185	NB	-1.095×10^2	2.293×10^2	2.229×10^2	2.230×10^2	0.15
			TT	-1.070×10^2	2.349×10^2	2.220×10^2	2.222×10^2	0.22
			Clinical	-2.335×10^2	4.826×10^2	4.730×10^2	4.731×10^2	0.00
			SEIR(2)	-1.070×10^2	2.297×10^2	2.200×10^2	2.202×10^2	0.63
			ST	-2.479×10^2	5.063×10^2	4.999×10^2	5.000×10^2	0.00
			SEIR(1)	-2.479×10^2	5.011×10^2	4.979×10^2	4.979×10^2	0.00
Mpox	Zaire	147	NB	-1.069×10^2	2.238×10^2	2.178×10^2	2.179×10^2	0.20
			TT	-1.074×10^2	2.348×10^2	2.228×10^2	2.231×10^2	0.01
			Clinical	-1.073×10^2	2.295×10^2	2.205×10^2	2.207×10^2	0.05
			SEIR(2)	-1.069×10^2	2.288×10^2	2.198×10^2	2.200×10^2	0.07
			ST	-1.074×10^2	2.248×10^2	2.188×10^2	2.189×10^2	0.12
			SEIR(1)	-1.074×10^2	2.198×10^2	2.168×10^2	2.169×10^2	0.34
SARS-CoV-1	Beijing	33	NB	-3.792×10^1	8.283×10^1	7.984×10^1	8.024×10^1	0.44
			TT	-3.795×10^1	8.988×10^1	8.390×10^1	8.532×10^1	0.03
			Clinical	-4.253×10^1	9.554×10^1	9.105×10^1	9.188×10^1	0.00
			SEIR(2)	-3.795×10^1	8.639×10^1	8.190×10^1	8.272×10^1	0.13
			ST	-4.433×10^1	9.565×10^1	9.266×10^1	9.306×10^1	0.00
			SEIR(1)	-4.433×10^1	9.216×10^1	9.066×10^1	9.079×10^1	0.00
		57	NB	-7.796×10^1	1.640×10^2	1.599×10^2	1.601×10^2	0.03
			TT	-7.380×10^1	1.638×10^2	1.556×10^2	1.564×10^2	0.23
			Clinical	-9.406×10^1	2.002×10^2	1.941×10^2	1.946×10^2	0.00
			SEIR(2)	-7.380×10^1	1.597×10^2	1.536×10^2	1.540×10^2	0.73
			ST	-9.961×10^1	2.073×10^2	2.032×10^2	2.034×10^2	0.00
			SEIR(1)	-9.961×10^1	2.033×10^2	2.012×10^2	2.013×10^2	0.00
Smallpox	Europe	32	NB	-6.882×10^1	1.446×10^2	1.416×10^2	1.421×10^2	0.29
			TT	-6.642×10^1	1.467×10^2	1.408×10^2	1.423×10^2	0.26
			Clinical	-7.366×10^1	1.577×10^2	1.533×10^2	1.542×10^2	0.00
			SEIR(2)	-6.732×10^1	1.450×10^2	1.406×10^2	1.415×10^2	0.39
			ST	-7.366×10^1	1.543×10^2	1.513×10^2	1.517×10^2	0.00
			SEIR(1)	-7.366×10^1	1.508×10^2	1.493×10^2	1.495×10^2	0.01
	West Midlands, UK	25	NB	-4.419×10^1	9.483×10^1	9.239×10^1	9.293×10^1	0.14
			TT	-4.426×10^1	1.014×10^2	9.652×10^1	9.852×10^1	0.01
			Clinical	-4.426×10^1	9.818×10^1	9.452×10^1	9.566×10^1	0.04
			SEIR(2)	-4.426×10^1	9.818×10^1	9.452×10^1	9.566×10^1	0.04
			ST	-4.426×10^1	9.496×10^1	9.252×10^1	9.307×10^1	0.13
			SEIR(1)	-4.426×10^1	9.174×10^1	9.052×10^1	9.069×10^1	0.44
Tuberculosis	Victoria, Australia	4234	NB	-4.304×10^3	8.625×10^3	8.613×10^3	8.613×10^3	1.00
			TT	-4.319×10^3	8.671×10^3	8.645×10^3	8.645×10^3	0.00
			Clinical	-4.421×10^3	8.867×10^3	8.848×10^3	8.848×10^3	0.00
			SEIR(2)	-4.319×10^3	8.663×10^3	8.643×10^3	8.643×10^3	0.00
			ST	-5.090×10^3	1.020×10^4	1.018×10^4	1.018×10^4	0.00
			SEIR(1)	-5.090×10^3	1.019×10^4	1.018×10^4	1.018×10^4	0.00

Table 2: Model performance. NB: Negative Binomial; TT: Two-type; ST: Single-typel.

Pathogen	Location	Model	Parameter	MLE	2.5%	25%	50%	75%	97.5%	ESS	$\hat{R}$
SARS-CoV-2	Batam	NB	R	0.91	0.61	0.83	0.99	1.18	1.71	3.907×10^3	1.00
		NB	k_{NB}	0.20	0.12	0.17	0.21	0.25	0.36	3.412×10^3	1.00
		TT	R	0.91	0.60	0.82	0.96	1.12	1.56	4.315×10^3	1.00
		TT	σ	1.00	0.01	0.17	0.41	0.70	0.97	4.088×10^3	1.00
		TT	c	0.20	0.11	0.19	0.24	0.30	0.44	3.421×10^3	1.00
		TT	ρ	0.04	0.01	0.03	0.04	0.06	0.11	3.741×10^3	1.00
	China	NB	R	0.40	0.35	0.38	0.40	0.42	0.46	4.139×10^3	1.00
		NB	k_{NB}	0.29	0.24	0.27	0.30	0.32	0.38	5.040×10^3	1.00
		TT	R	0.40	0.35	0.38	0.40	0.42	0.46	4.621×10^3	1.00
		TT	σ	0.00	0.01	0.08	0.20	0.42	0.92	3.326×10^3	1.00
		TT	c	0.20	0.10	0.16	0.20	0.24	0.35	3.153×10^3	1.00
		TT	ρ	0.12	0.06	0.08	0.10	0.11	0.14	3.035×10^3	1.00
		NB	R	0.69	0.64	0.67	0.69	0.71	0.74	4.489×10^3	1.00
		NB	k_{NB}	0.75	0.64	0.71	0.75	0.79	0.89	4.675×10^3	1.00
		TT	R	0.69	0.64	0.67	0.69	0.71	0.75	4.913×10^3	1.00
		TT	σ	0.14	0.03	0.14	0.27	0.49	0.93	2.226×10^3	1.00
		TT	c	0.08	0.04	0.09	0.12	0.16	0.26	2.159×10^3	1.00
		TT	ρ	0.22	0.16	0.20	0.22	0.25	0.33	1.925×10^3	1.00
	Hong Kong	NB	R	0.58	0.47	0.55	0.60	0.65	0.75	3.958×10^3	1.00
		NB	k_{NB}	0.43	0.29	0.38	0.43	0.50	0.68	4.302×10^3	1.00
		TT	R	0.58	0.47	0.54	0.60	0.65	0.77	4.298×10^3	1.00
		TT	σ	0.00	0.01	0.12	0.31	0.62	0.96	3.449×10^3	1.00
		TT	c	0.12	0.05	0.11	0.16	0.24	0.48	3.102×10^3	1.00
		TT	ρ	0.16	0.07	0.11	0.14	0.17	0.26	3.458×10^3	1.00
	India	NB	R	0.48	0.48	0.48	0.48	0.49	0.49	4.932×10^3	1.00
		NB	k_{NB}	0.51	0.49	0.50	0.51	0.51	0.52	3.878×10^3	1.00
		TT	R	0.48	0.48	0.48	0.48	0.49	0.49	3.702×10^3	1.00
		TT	σ	0.10	0.04	0.08	0.11	0.13	0.20	2.364×10^3	1.00
		TT	c	0.10	0.09	0.10	0.10	0.10	0.11	2.955×10^3	1.00
		TT	ρ	0.17	0.15	0.16	0.17	0.17	0.18	2.430×10^3	1.00
	Jakarta	NB	R	0.91	0.74	0.86	0.93	1.02	1.19	4.172×10^3	1.00
		NB	k_{NB}	0.06	0.05	0.06	0.06	0.06	0.07	4.392×10^3	1.00
		TT	R	0.91	0.76	0.86	0.92	0.98	1.12	3.414×10^3	1.00
		TT	σ	0.00	0.00	0.04	0.08	0.14	0.31	1.805×10^3	1.00
		TT	c	0.18	0.14	0.16	0.17	0.18	0.20	2.253×10^3	1.00
		TT	ρ	0.00	0.00	0.00	0.00	0.00	0.00	1.876×10^3	1.00
	South Korea (a)	NB	R	0.81	0.64	0.76	0.82	0.90	1.07	4.855×10^3	1.00
		NB	k_{NB}	0.23	0.17	0.21	0.23	0.26	0.31	4.199×10^3	1.00
		TT	R	0.81	0.62	0.75	0.83	0.92	1.15	3.573×10^3	1.00
		TT	σ	0.00	0.00	0.03	0.07	0.16	0.72	3.834×10^3	1.00
		TT	c	0.06	0.03	0.06	0.08	0.10	0.16	3.102×10^3	1.00
		TT	ρ	0.06	0.03	0.05	0.06	0.07	0.10	3.030×10^3	1.00
	South Korea (b)	NB	R	0.68	0.63	0.66	0.68	0.70	0.74	4.771×10^3	1.00
		NB	k_{NB}	0.85	0.70	0.80	0.85	0.91	1.05	4.065×10^3	1.00
		TT	R	0.68	0.62	0.66	0.68	0.71	0.76	5.040×10^3	1.00
		TT	σ	0.35	0.10	0.29	0.46	0.68	0.97	2.156×10^3	1.00
		TT	c	0.03	0.01	0.03	0.04	0.06	0.11	1.550×10^3	1.00
		TT	ρ	0.14	0.09	0.13	0.16	0.19	0.26	1.722×10^3	1.00

Pathogen	Location	Model	Parameter	MLE	2.5%	25%	50%	75%	97.5%	ESS	$\hat{R}$
EBV	Guinea	NB	R	0.95	0.68	0.88	1.01	1.16	1.58	3.566×10^3	1.00
		NB	k_{NB}	0.18	0.12	0.16	0.18	0.21	0.28	4.019×10^3	1.00
		TT	R	0.95	0.68	0.87	0.98	1.10	1.43	4.395×10^3	1.00
		TT	σ	0.00	0.01	0.10	0.25	0.53	0.95	3.764×10^3	1.00
		TT	c	0.31	0.15	0.22	0.27	0.31	0.42	3.103×10^3	1.00
		TT	ρ	0.03	0.00	0.02	0.03	0.04	0.07	3.038×10^3	1.00
MERS-CoV	Korea	NB	R	0.94	0.54	0.87	1.13	1.52	2.81	3.820×10^3	1.00
		NB	k_{NB}	0.03	0.02	0.03	0.03	0.04	0.05	3.644×10^3	1.00
		TT	R	0.94	0.51	0.84	1.11	1.47	2.51	2.782×10^3	1.00
		TT	σ	0.00	0.01	0.08	0.23	0.51	0.96	3.758×10^3	1.00
		TT	c	0.03	0.02	0.03	0.04	0.05	0.08	3.174×10^3	1.00
		TT	ρ	0.00	0.00	0.00	0.00	0.01	0.01	2.802×10^3	1.00
Mpox	Zaire	NB	R	0.32	0.24	0.30	0.34	0.38	0.48	3.765×10^3	1.00
		NB	k_{NB}	0.58	0.27	0.47	0.64	0.90	1.76	3.259×10^3	1.00
		TT	R	0.32	0.24	0.30	0.33	0.37	0.46	5.339×10^3	1.00
		TT	σ	0.00	0.01	0.16	0.39	0.67	0.97	4.522×10^3	1.00
		TT	c	0.00	0.04	0.26	0.48	0.69	0.95	3.884×10^3	1.00
		TT	ρ	0.00	0.02	0.16	0.32	0.60	0.95	3.904×10^3	1.00
SARS-CoV-1	Beijing	NB	R	0.94	0.52	0.87	1.16	1.54	2.80	3.184×10^3	1.00
		NB	k_{NB}	0.17	0.07	0.13	0.18	0.25	0.45	2.986×10^3	1.00
		TT	R	0.94	0.53	0.82	1.03	1.32	2.06	4.397×10^3	1.00
		TT	σ	0.00	0.01	0.16	0.39	0.69	0.97	4.795×10^3	1.00
		TT	c	0.33	0.08	0.20	0.28	0.36	0.57	3.585×10^3	1.00
		TT	ρ	0.02	0.00	0.02	0.04	0.08	0.22	3.300×10^3	1.00
		NB	R	1.63	0.95	1.40	1.71	2.11	3.29	3.622×10^3	1.00
		NB	k_{NB}	0.16	0.09	0.13	0.16	0.20	0.28	3.627×10^3	1.00
		TT	R	1.63	0.84	1.33	1.72	2.22	3.69	2.419×10^3	1.00
		TT	σ	0.00	0.01	0.17	0.41	0.69	0.97	3.619×10^3	1.00
		TT	c	0.07	0.03	0.06	0.08	0.11	0.20	2.589×10^3	1.00
		TT	ρ	0.03	0.01	0.02	0.03	0.04	0.07	2.790×10^3	1.00
Smallpox	Europe	NB	R	3.19	1.77	2.50	2.97	3.56	5.09	3.431×10^3	1.00
		NB	k_{NB}	0.37	0.20	0.30	0.38	0.47	0.71	3.589×10^3	1.00
		TT	R	3.19	1.68	2.39	2.87	3.46	4.87	3.387×10^3	1.00
		TT	σ	1.00	0.02	0.24	0.51	0.76	0.97	3.491×10^3	1.00
		TT	c	0.25	0.12	0.21	0.28	0.36	0.57	3.246×10^3	1.00
		TT	ρ	0.07	0.03	0.06	0.08	0.11	0.21	4.117×10^3	1.00
	West Midlands, UK	NB	R	1.68	1.05	1.46	1.73	2.05	2.88	3.260×10^3	1.00
		NB	k_{NB}	0.82	0.32	0.59	0.81	1.12	2.12	3.148×10^3	1.00
		TT	R	1.68	1.07	1.46	1.69	1.98	2.79	3.577×10^3	1.00
		TT	σ	0.00	0.02	0.20	0.46	0.72	0.98	3.948×10^3	1.00
		TT	c	0.00	0.04	0.34	0.58	0.76	0.97	3.665×10^3	1.00
		TT	ρ	0.00	0.02	0.18	0.40	0.67	0.96	3.051×10^3	1.00
Tuberculosis	Victoria, Australia	NB	R	0.76	0.70	0.74	0.76	0.78	0.83	4.142×10^3	1.00
		NB	k_{NB}	0.16	0.14	0.15	0.16	0.16	0.17	4.794×10^3	1.00
		TT	R	0.76	0.70	0.74	0.76	0.78	0.82	3.798×10^3	1.00
		TT	σ	0.00	0.00	0.03	0.05	0.09	0.18	2.514×10^3	1.00
		TT	c	0.25	0.21	0.23	0.24	0.25	0.27	2.660×10^3	1.00
		TT	ρ	0.03	0.02	0.03	0.03	0.03	0.04	2.865×10^3	1.00

Table 3: Parameter estimates. NB: Negative Binomial; TT: Two-type. R: population mean reproductive number; k_{NB}: dispersion parameter; σ: relative transmissibility of first serial compartment; c: superspreader fraction; ρ: relative transmissibility of subspreaders.

Pathogen	Location	n_{obs}	Model	$\ell_{\max}$	BIC	AIC	AIC$_c$	w
EBV	Guinea	152	NB	-1.772×10^2	3.644×10^2	3.584×10^2	3.585×10^2	0.93
			Three-type	-1.770×10^2	3.841×10^2	3.660×10^2	3.665×10^2	0.02
			SEIR(3)	-1.770×10^2	3.791×10^2	3.640×10^2	3.644×10^2	0.05
MERS-CoV	Korea	185	NB	-1.095×10^2	2.293×10^2	2.229×10^2	2.230×10^2	0.13
			Three-type	-1.047×10^2	2.407×10^2	2.214×10^2	2.219×10^2	0.22
			SEIR(3)	-1.047×10^2	2.355×10^2	2.194×10^2	2.197×10^2	0.65
Mpox	Zaire	147	NB	-1.069×10^2	2.238×10^2	2.178×10^2	2.179×10^2	0.94
			Three-type	-1.069×10^2	2.436×10^2	2.257×10^2	2.263×10^2	0.01
			SEIR(3)	-1.069×10^2	2.387×10^2	2.237×10^2	2.241×10^2	0.04
SARS-CoV-1	Beijing	33	NB	-3.792×10^1	8.283×10^1	7.984×10^1	8.024×10^1	0.97
			Three-type	-3.780×10^1	9.657×10^1	8.759×10^1	9.083×10^1	0.00
			SEIR(3)	-3.780×10^1	9.308×10^1	8.559×10^1	8.782×10^1	0.02
		57	NB	-7.796×10^1	1.640×10^2	1.599×10^2	1.601×10^2	0.28
			Three-type	-7.380×10^1	1.719×10^2	1.596×10^2	1.613×10^2	0.16
			SEIR(3)	-7.380×10^1	1.678×10^2	1.576×10^2	1.588×10^2	0.56
SARS-CoV-2	Batam	89	NB	-1.035×10^2	2.159×10^2	2.109×10^2	2.111×10^2	0.93
			Three-type	-1.031×10^2	2.330×10^2	2.181×10^2	2.191×10^2	0.02
			SEIR(3)	-1.031×10^2	2.286×10^2	2.161×10^2	2.168×10^2	0.05
	China	1178	NB	-9.444×10^2	1.903×10^3	1.893×10^3	1.893×10^3	0.60
			Three-type	-9.421×10^2	1.927×10^3	1.896×10^3	1.896×10^3	0.11
			SEIR(3)	-9.421×10^2	1.920×10^3	1.894×10^3	1.894×10^3	0.29
		2048	NB	-2.329×10^3	4.674×10^3	4.663×10^3	4.663×10^3	0.00
			Three-type	-2.318×10^3	4.682×10^3	4.648×10^3	4.648×10^3	0.31
			SEIR(3)	-2.318×10^3	4.675×10^3	4.647×10^3	4.647×10^3	0.69
	Hong Kong	290	NB	-2.950×10^2	6.013×10^2	5.939×10^2	5.940×10^2	0.64
			Three-type	-2.928×10^2	6.196×10^2	5.976×10^2	5.979×10^2	0.09
			SEIR(3)	-2.928×10^2	6.139×10^2	5.955×10^2	5.958×10^2	0.26
	India	88527	NB	-8.199×10^4	1.640×10^5	1.640×10^5	1.640×10^5	0.00
			Three-type	-8.147×10^4	1.630×10^5	1.630×10^5	1.630×10^5	0.83
			SEIR(3)	-8.148×10^4	1.630×10^5	1.630×10^5	1.630×10^5	0.17
	Jakarta	1199	NB	-1.013×10^3	2.041×10^3	2.031×10^3	2.031×10^3	0.00
			Three-type	-1.002×10^3	2.046×10^3	2.016×10^3	2.016×10^3	0.27
			SEIR(3)	-1.002×10^3	2.039×10^3	2.014×10^3	2.014×10^3	0.73
	South Korea (a)	344	NB	-3.823×10^2	7.763×10^2	7.686×10^2	7.687×10^2	0.00
			Three-type	-3.718×10^2	7.787×10^2	7.557×10^2	7.559×10^2	0.26
			SEIR(3)	-3.718×10^2	7.729×10^2	7.537×10^2	7.538×10^2	0.74
	South Korea (b)	1401	NB	-1.588×10^3	3.190×10^3	3.179×10^3	3.179×10^3	0.00
			Three-type	-1.571×10^3	3.185×10^3	3.154×10^3	3.154×10^3	0.43
			SEIR(3)	-1.572×10^3	3.179×10^3	3.153×10^3	3.153×10^3	0.57
Smallpox	Europe	32	NB	-6.882×10^1	1.446×10^2	1.416×10^2	1.421×10^2	0.90
			Three-type	-6.730×10^1	1.554×10^2	1.466×10^2	1.500×10^2	0.02
			SEIR(3)	-6.732×10^1	1.520×10^2	1.446×10^2	1.469×10^2	0.08
	West Midlands, UK	25	NB	-4.419×10^1	9.483×10^1	9.239×10^1	9.293×10^1	0.99
			Three-type	-4.426×10^1	1.078×10^2	1.005×10^2	1.052×10^2	0.00
			SEIR(3)	-4.426×10^1	1.046×10^2	9.852×10^1	1.017×10^2	0.01
Tuberculosis	Victoria, Australia	4234	NB	-4.304×10^3	8.625×10^3	8.613×10^3	8.613×10^3	0.09
			Three-type	-4.299×10^3	8.649×10^3	8.611×10^3	8.611×10^3	0.24
			SEIR(3)	-4.299×10^3	8.640×10^3	8.609×10^3	8.609×10^3	0.67

Table 4: **Extended model performance.** Performance of the uncosntrained three-type model and SEIR(3) model, in comparison with the negative binomial benchmark.

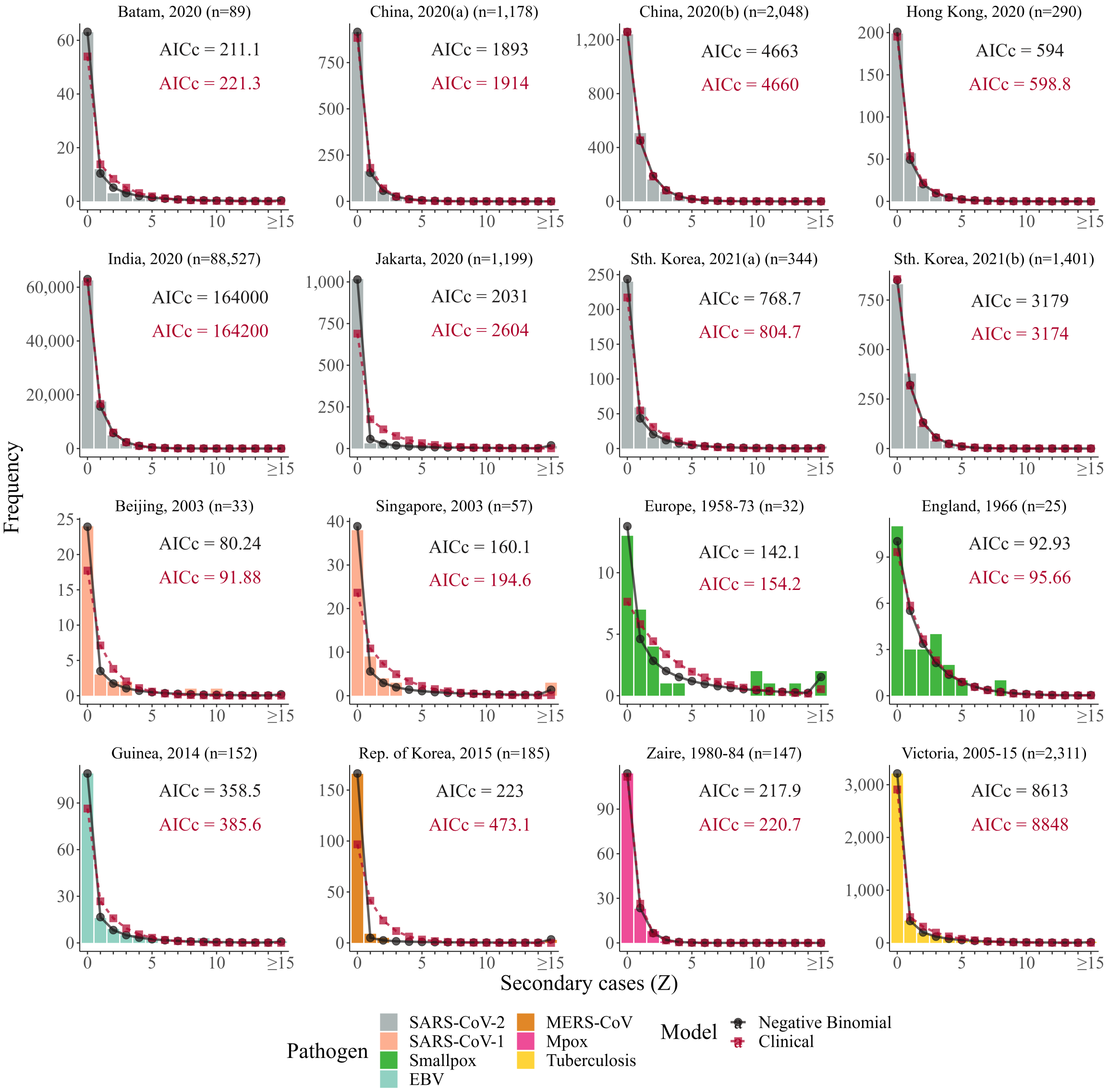

Figure 6: **Model fits to secondary case counts**. Secondary case count distributions for 16 outbreaks along with the best-fitting (according to maximum likelihood) negative binomial (blue circles and solid line) and clinical (red square) model predictions and their corresponding corrected Akaike information criteria (AIC_c). Each offspring distribution has been colored according to the corresponding pathogen: SARS-CoV-2 (gray); SARS-CoV-1 (salmon); smallpox (green); EBV (light blue); MERS-CoV (brown); Mpox (pink); and tuberculosis (yellow). Each panel is labelled by the location, year and size of each outbreak.

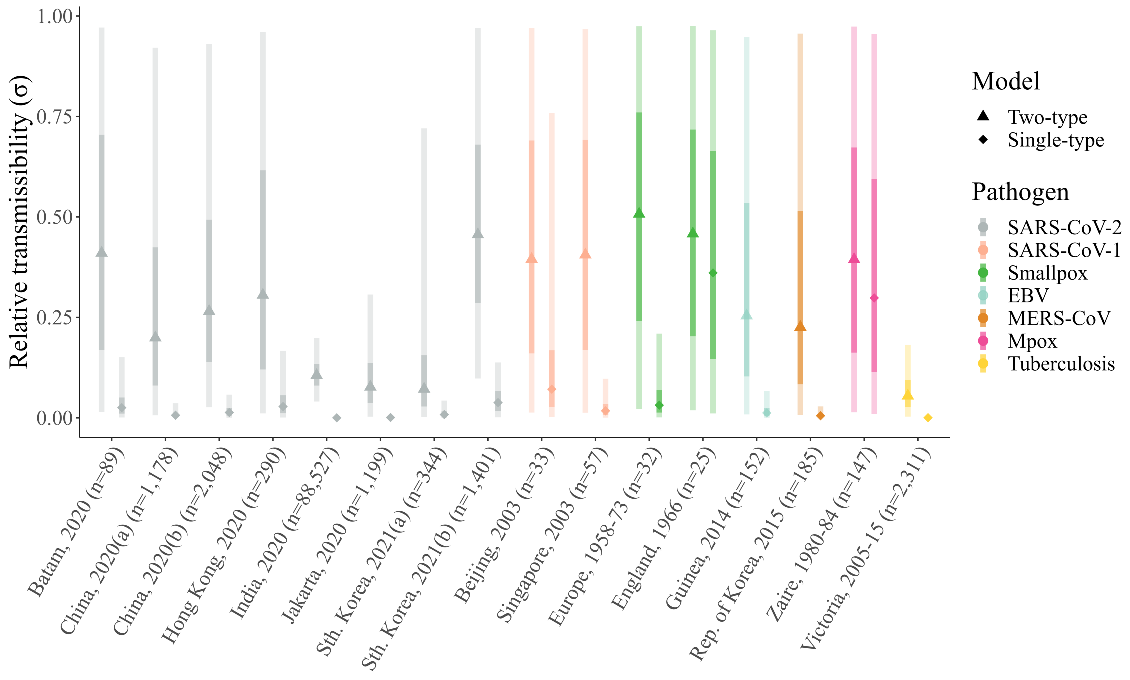

Figure 7: **Relative transmissibility of first and second serial compartments**. Parameter estimates for the relative transmissibility of the first and second serial compartments, σ, for the two-type and single-type models. Markers indicate the median posterior estimate for σ whilst the dark and light shaded bands give the 25-75% and 2.5-97.5% credible intervals, respectively. Each marker and interval is colored according to the corresponding pathogen: SARS-CoV-2 (gray); SARS-CoV-1 (salmon); smallpox (green); EBV (light blue); MERS-CoV (brown); Mpox (pink); and tuberculosis (yellow). Each outbreak is labelled according to location, year and size.

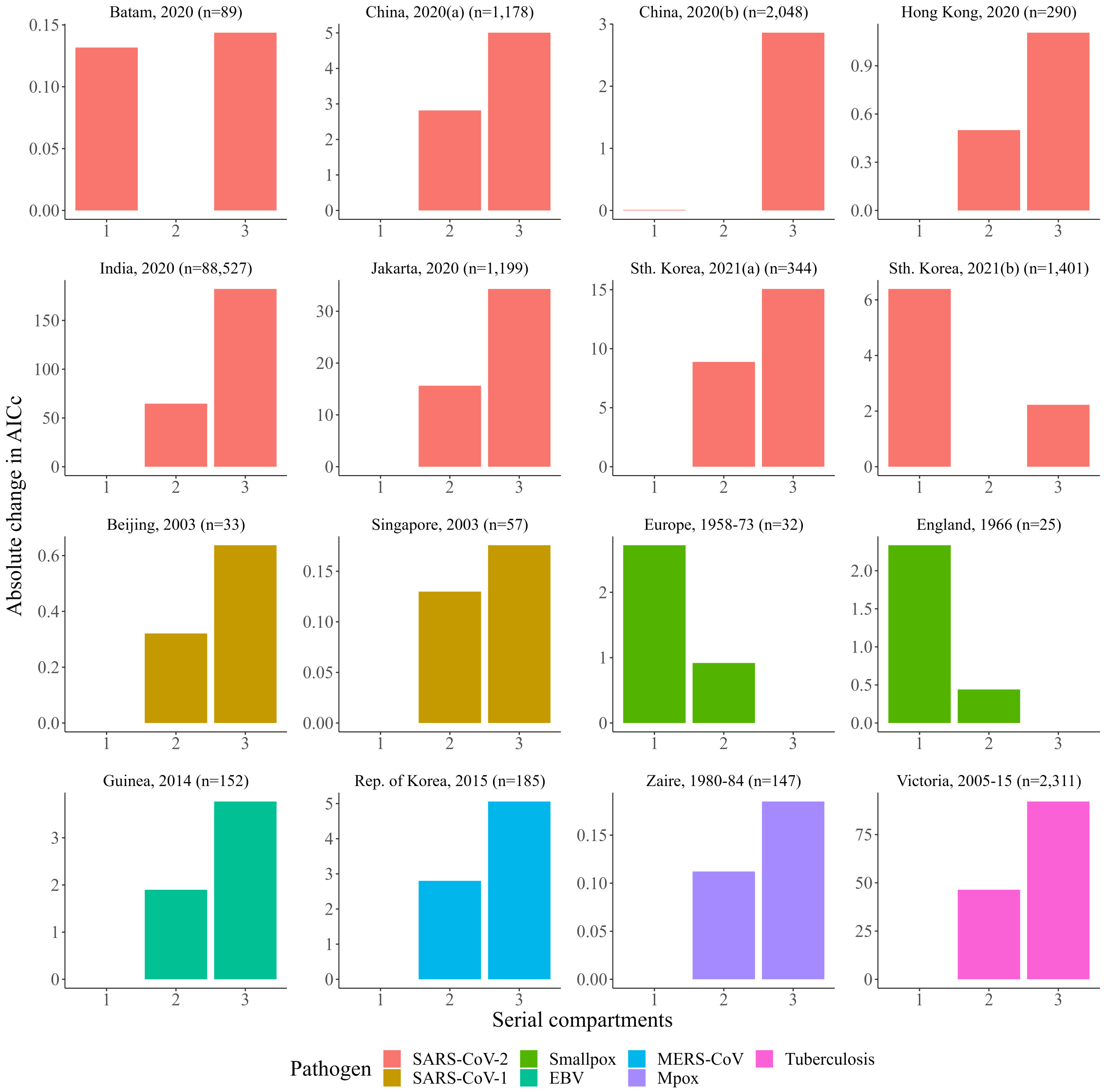

Figure 8: **Performance sensitivity to serial structure**. Change in corrected Akaike information criteria (AIC_c) for the SEIR(2) model for different numbers of actively infectious serial compartments, relative to the minimum AIC_c value. Each bar is colored according to the corresponding pathogen: SARS-CoV-2 (gray); SARS-CoV-1 (salmon); smallpox (green); EBV (light blue); MERS-CoV (brown); Mpox (pink); and tuberculosis (yellow). Each outbreak is labelled according to location, year and size.

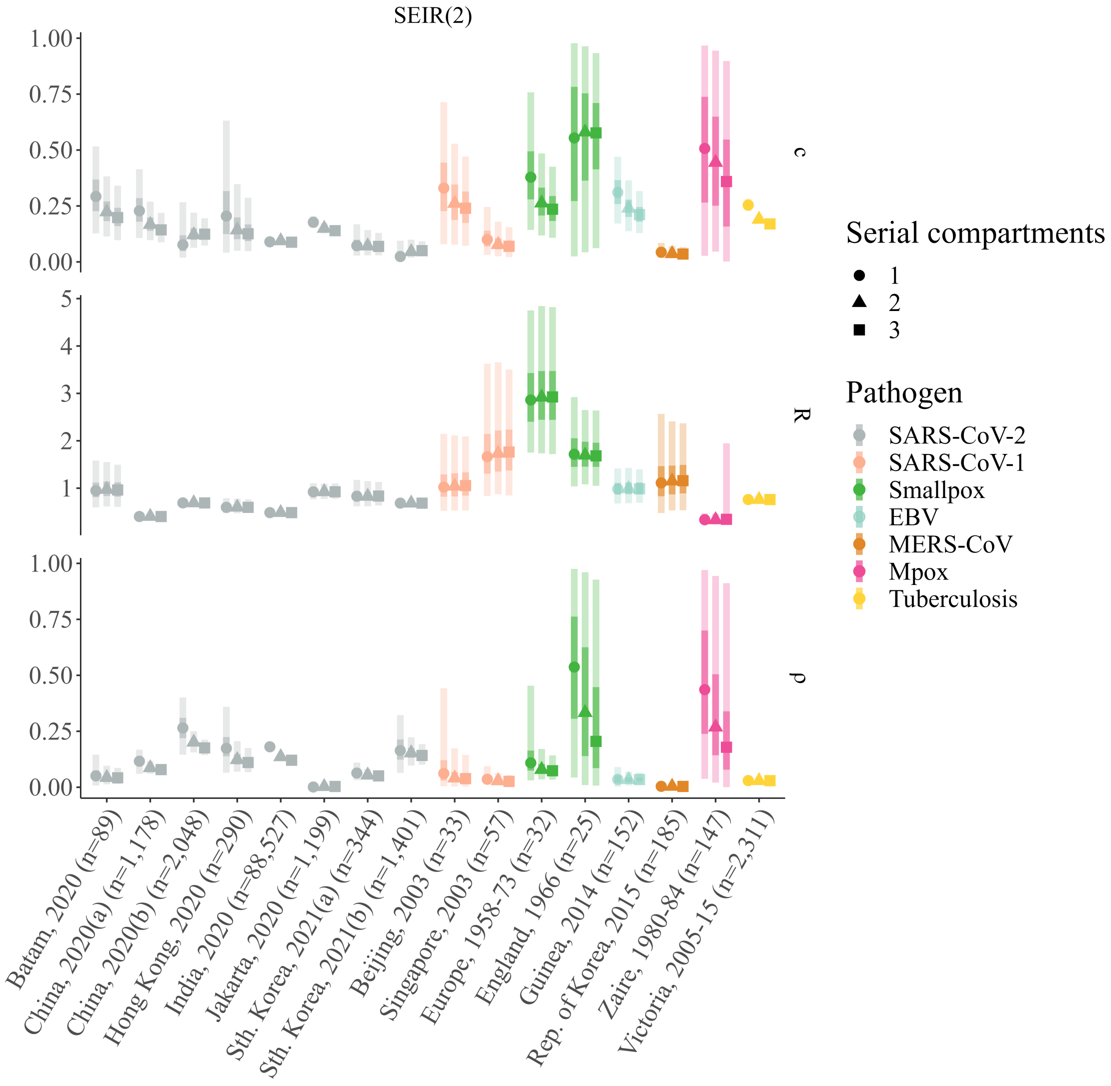

Figure 9: **Sensitivity to serial structure**. Parameter estimates for the mean reproduction number, R, the superspreader fraction c, and the relative transmissibility of subspreaders, ρ, for varying numbers of actively infectious serial compartments with the SEIR(2) model. Each marker and interval is colored according to the corresponding pathogen: SARS-CoV-2 (gray); SARS-CoV-1 (salmon); smallpox (green); EBV (light blue); MERS-CoV (brown); Mpox (pink); and tuberculosis (yellow). Each outbreak is labelled according to location, year and size.
